# Impact of multiple different high-fat diets on metabolism, inflammatory markers, dysbiosis, and liver histology: study on NASH rat model induced diet

**DOI:** 10.12688/f1000research.129645.1

**Published:** 2023-02-15

**Authors:** Syifa Mustika, Dewi Santosaningsih, Dian Handayani, Achmad Rudijanto

**Affiliations:** 1 Medical Science, Universitas Brawijaya, Malang, Jl. Veteran, 65145, Indonesia; 2 Clinical Microbiology, Universitas Brawijaya, Malang, Jl. Veteran, 65145, Indonesia; 3 Nutrition, Universitas Brawijaya, Malang, Jl. Veteran, 65145, Indonesia; 4 Internal Medicine, Universitas Brawijaya, Malang, Jl. Veteran, 65145, Indonesia

**Keywords:** diet, non-alcoholic steatohepatitis, Rattus norvegicus strain Wistar

## Abstract

**Background:** The spectrum of non-alcoholic fatty liver disease (NAFLD), known as non-alcoholic steatohepatitis (NASH), can lead to advanced liver disease. It is known that a variety of diets play a significant role in the development of NAFLD/NASH. The goal of this study was to determine the most appropriate composition of diet to induce NASH in an animal model.

**Methods:** This research used *Rattus norvegicus* strain Wistar (n=27), which were divided into four groups and given each diet for 12 weeks: normal diet (ND, n=7), high-fat diet (HFD, n=6), western diet (WD, n=7), and high-fat-high-fructose diet (HFHFD, n=7). Subjects were documented for body weight. Blood samples were taken for biochemical analysis: l *ow-density lipoprotein* (LDL), triglyceride, alanine aminotransferase (ALT), aspartate aminotransferase (AST), alkaline phosphatase (ALP), hepatic lipase, tumor necrosis factor-alpha (TNF-α), interleukin-6 (IL-6), and lipopolysaccharide (LPS). Feces were taken for *short-chain fatty acid* (SCFA) analysis. Liver histology was analyzed using NAS (NAFLD activity score). The comparison test was carried out using the one-way ANOVA or Kruskal–Wallis test.

**Results:** The highest average body weight was in the WD group (346.14 g). Liver enzymes, LDL, triglyceride, propionic acid, and acetic acid in each group were not significantly different. TNF-α, IL-6, and hepatic lipase were significant (p = 0.000; p = 0.000; p = 0.004) and the highest was in the HFD group. Butyrate level was significant (p = 0.021) and the least was in the HFHFD group (4.77 mMol/g). Only WD and HFHFD had an NAS ≥ 5 (14% and 14%). The highest percentage of borderline NAS was found in WD (57%).

**Conclusions:** The HFD group showed significant liver inflammation but did not produce NASH histologically, whereas the WD and HFHFD groups had the potential to develop NASH because the diets affected metabolic and inflammatory parameters as well as liver histology.

## Introduction

Non-alcoholic fatty liver disease (NAFLD) is becoming a common medical problem because of its high incidence and treatment complexity. According to the most recent epidemiology, NAFLD has become the second most common liver disease after viral hepatitis, with a 20–30% incidence rate, and obesity rates of up to 57.74% in the global population. ^1^

The NAFLD subtype, non-alcoholic steatohepatitis (NASH), has become a major public health concern. ^2^ NASH is defined via liver biopsy as the presence of ≥5% hepatic steatosis and inflammation with hepatocyte injury ( *e.g.,* ballooning), with or without any fibrosis. It is a potentially progressive liver disease that can lead to cirrhosis. ^3^ Risk factors for the development of NASH include excessive calorie-dense food intake, lack of physical activity and exercise, and genetic susceptibility. ^4^

Poor dietary habits may induce NASH, directly by affecting hepatic triglyceride accumulation and antioxidant activity, and indirectly by impairing insulin sensitivity and fat metabolism. ^5^ According to a previous study, there will be a 33.5% increase in the total prevalence of NAFLD by 2030. This condition is associated with a significant increased incidence of NASH complications, such as decompensated cirrhosis (168%), hepatocellular carcinoma (137%), and liver-related mortality (178%). ^6^

The current problem with NAFLD/NASH is that there is no universally accepted treatment as standard. Furthermore, clinical research on the NASH/NAFLD mechanism is limited by ethical considerations when using humans as research subjects, as it involves obtaining tissue samples from patients. ^7^ Besides that, the development of NASH in humans can take a long time, up to several decades. Therefore, research related to NASH requires an appropriate experimental animal model to represent NASH.

Diet can facilitate the development of NAFLD/NASH. The high-fat diet (HFD), western diet (WD), and high-fat-high-fructose diet (HFHFD) are the types of diet used to induce NASH. ^8^^,^^9^ Of the various diets, there is no standard composition to describe the condition of NASH. Based on the problems above, we need to create an animal model of NASH based on diet intervention. This study is aimed to determine the most representative diet for inducing NASH in the *Rattus norvegicus* Wistar strain.

## Methods

### Animals and diet

The Ethical Committee of the Faculty of Medicine, Universitas Brawijaya reviewed and approved all procedures (No. 66/EC/KEPK/02/2021). A total of 27 male Wistar rats were obtained from Universitas Gadjah Mada. Rat inclusion criteria included: male rat with shiny white fur, healthy, active, and had normal behavior; about 8-12 weeks old; the average body weight was 150-180 grams. Exclusion criteria included: the appearance of dull fur, loss and baldness; less or inactive activity; rats that during the study did not want to eat; weight loss >10% after adaptation period; disabled, sick and/or dead rat. This research used the refinement principle for ensuring the welfare of experimental animals until the end of the study to minimize pain and discomfort. We provided food and drink regularly every day with a certain type of diet according to the type of treatment. Cage maintenance, cage cleaning, and wood husk replacement were carried out every day with attention to light, temperature, and humidity. We monitored and evaluated the rats’ condition every day and placed them individually in each cage. Before being treated, the rats were acclimatized for two weeks, given a standard diet, and placed inside cages at the Pharmacology Laboratory, Faculty of Medicine, Universitas Brawijaya. The Wistar rats were randomly assigned using a table of random numbers, then categorized into four groups: normal diet (ND) (67% carbohydrate, 21% protein, 7% fat, 5% fiber); HFD (67.1% carbohydrate, 16.5% fat, 16.4% protein), WD (52% carbohydrate, 16.1% protein, 31.7% fat), and HFHFD (41.5% carbohydrate, 10.3% fat, 10.2% protein, 38% fructose). All diets were given for 12 weeks. All four groups were euthanized with ketamine–xylazine intravenously to relieve pain on the same day before surgery was performed. ^10^ The entire liver was taken out and weighed. For further analysis, the livers were either collected and stored at −20°C or fixed in 10% paraformaldehyde.

### Biochemical measurements and assays

Rat serum was used to analyze biochemical parameters in the Clinical Pathology Laboratory, Universitas Brawijaya, Indonesia. ^11^ Serum alanine aminotransferase (ALT), aspartate aminotransferase (AST), and alkaline phosphatase (ALP) were analyzed chemically using colorimetric analysis (ADVIA 2400 Clinical Chemistry System (Siemens, Germany). Serum hepatic lipase, tumor necrosis factor-alpha (TNF-α), interleukin-6 (IL-6), and lipopolysaccharide (LPS) were analyzed with the sandwich enzyme-linked immunosorbent assay (ELISA) method. ^12^

### Fecal sample preparation and SCFA measurement

A total of 0.5 gram fecal samples from the colon were collected, labelled, and placed into container tubes. These samples were immediately stored at −40°C until the analysis day. At the time of analysis, 0.2 g of the fecal sample supernatant poured into a 2 mL microtube and then added with sterile aquabidest water for injection. This suspension underwent 20 minutes of sonification, followed by centrifugation (14,000 rpm, 4°C, 10 min). The second centrifugation step (1,000 rpm, 4°C, 10 min) was performed while the natant was discarded. The final supernatant was injected to a gas chromatography (Shimadzu, GC-2010 Plus, Kyoto, Japan). Fecal pH measurement was used using pH meter (pH Spear Eutech, Eutech Instruments, Paisley, United Kingdom). ^13^ This procedure was performed at the Food Technology and Agricultural Products Laboratory, Universitas Gadjah Mada, Indonesia.

### Histopathology assessment of NAS

The liver was sliced, fixed with 10% buffered formalin, embedded in paraffin, and stained with hematoxylin–eosin (HE) stain at a thickness of 5 μm. ^11^ The sample preparation was performed at the Anatomical Pathology Laboratory of Universitas Brawijaya, Indonesia. Liver histopathology was used to find the NAFLD Activity Score (NAS). Three parameters (steatosis score 0–3; lobules inflammation score 0–3; ballooning score 0–2) were used to know NAFLD staging. Scores of 0–2 are defined non NASH, scores of 3–4 are defined as borderline, while scores ≥ 5 are considered diagnostic of NASH. ^14^

### Statistical analysis

Data were presented as the mean ± standard deviation and were analyzed with SPSS 25.0 (RRID:SCR_002865) for Windows. A one way ANOVA was carried out when the data were normally distributed and then continued with the Tukey Honest Significant Difference (HSD) *post hoc* test if the data were significant. The Kruskal–Wallis test was used when the data distribution was not normal. If the results were significant then the Mann Whitney test was performed. When p <0.05, data were considered significant.

**Figure 1.**
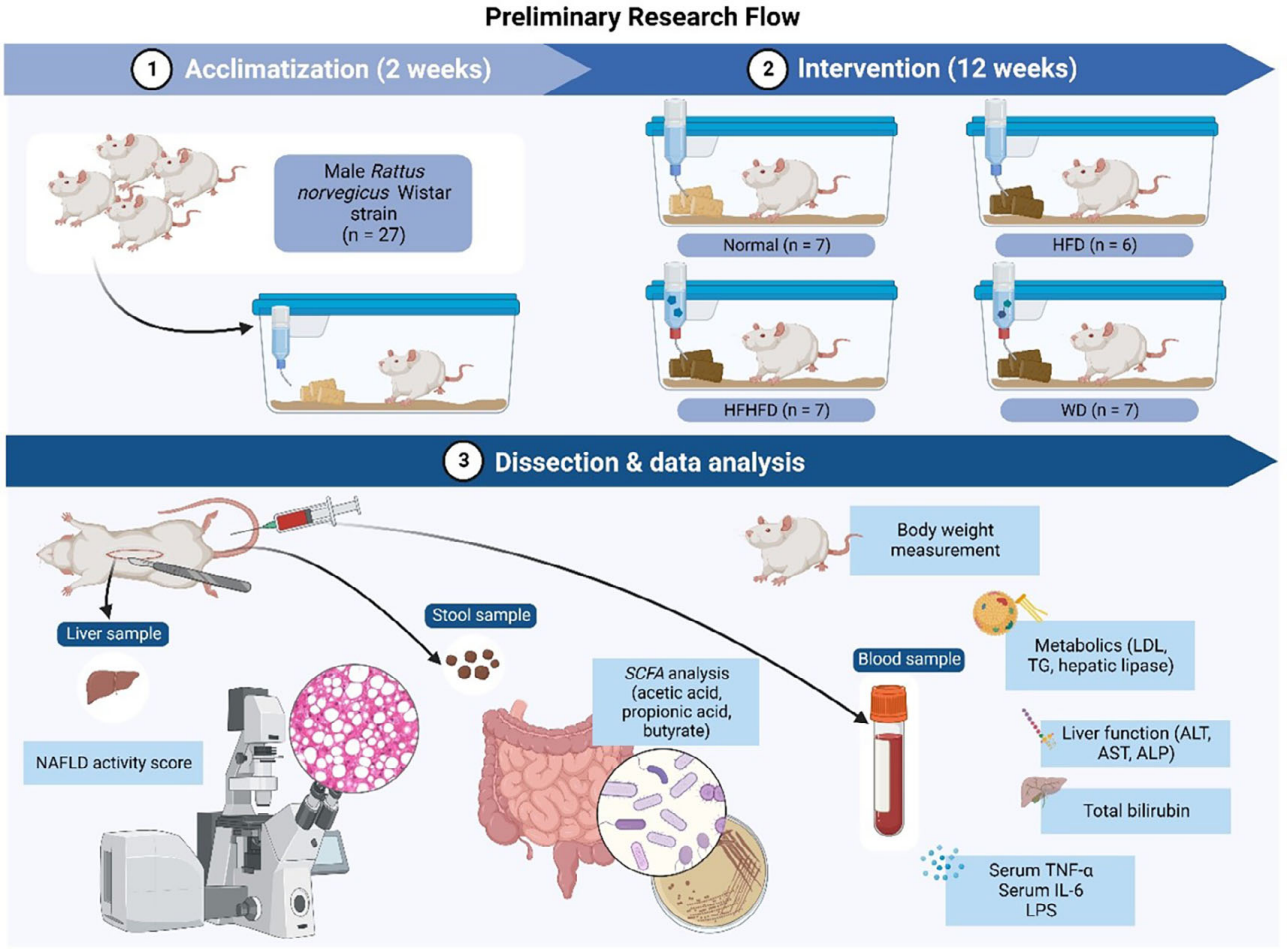
Research flow.

The research flow ( Figure 1) consisted of: 1). *Rattus norvegicus* acclimatization for two weeks; 2). Several diets intervention (ND, HFD, WD, HFHFD) for 12 weeks; 3). Dissection and data analysis at the end process. Several samples such as liver, stool, and blood were obtained for further analysis.

## Results

This research was done by following the method and research flow that has been explained above. The data of baseline characteristics and biochemical parameters of the rats were gathered and analyzed using the one-way ANOVA test and Kruskal–Wallis test. The need of this analysis was to obtain the data samples of baseline characteristics and biochemical parameters of rats after several diets intervention (ND, HFD, WD, HFHFD) for 12 weeks ( Table 1).

**Table 1.** Baseline characteristics and biochemical parameters of rats.

Parameters	ND (Mean ± SD)	HFD (Mean ± SD)	WD (Mean ± SD)	HFHFD (Mean ± SD)	p
Metabolic					
Body weight (g)	294.57 ± 8.73	326.67 ± 23.69	346.14 ± 55.60	285.43 ± 63.87	0.154 ^#^
LDL (mg/dL)	31.33 ± 9.35	35.17 ± 10.92	24.19 ± 7.48	20.25 ± 9.01	0.056 ^*^
Triglyceride (mg/dL)	79.71 ± 27.88	72.17 ± 33.58	125.14 ± 78.10	82.43 ± 39.70	0.354 ^#^
Hepatic lipase (ng/L)	4417.86 ± 430.54	7249.83 ± 1372.31	3637.86 ± 1210.17	4257.08 ± 1046.45	0.004 ^#^
Inflammatory and liver injury					
AST (U/L)	113.29 ± 25.20	104.50 ± 42.04	72.86 ± 15.30	88.14 ± 29.18	0.11 ^*^
ALT (U/L)	48.00 ± 12.01	57.00 ± 20.45	39.43 ± 12.23	44.57 ± 17.80	0.337 ^*^
Total bilirubin (mg/dL)	0.44 ± 0.07	0.48 ± 0.04	0.38 ± 0.08	0.47 ± 0.04	0.112 ^#^
TNF-α (ng/mL)	133.64 ± 20.01	352.88 ± 67.88	243.83 ± 25.07	173.57 ± 41.44	0.000 ^*^
IL-6 (ng/mL)	3.91 ± 0.64	20.39 ± 7.62	19.36 ± 3.03	9.70 ± 1.91	0.000 ^#^
ALP (U/L)	57.57 ± 9.76	279.50 ± 202.10	485.86 ± 84.26	120.86 ± 30.12	0.000 ^#^
Microbial dysbiosis					
LPS (EU/L)	322.70 ± 65.67	284.40 ± 65.55	250.06 ± 30.31	172.68 ± 51.71	0.001 ^*^
Acetic acid (mMol/g)	66.43 ± 7.27	61.85 ± 13.01	68.18 ± 23.82	54.60 ± 9.79	0.419 ^#^
Propionic acid (mMol/g)	21.28 ± 4.65	25.19 ± 5.06	29.69 ± 11.81	28.03 ± 8.16	0.316 ^#^
Butyrate (mMol/g)	10.56 ± 3.83	6.10 ± 2.68	7.47 ± 5.09	4.77 ± 1.48	0.021 ^#^
Liver histology scoring					
NAS	-	2.16 ± 0.69	3.42 ± 1.29	2.85 ± 1.24	0.209 ^*^

ND: normal diet; HFD: high-fat diet; WD: western diet; HFHFD: high-fat-high-fructose diet.

Significant with p < 0.05.

* One-way ANOVA test.

^#^ Kruskal–Wallis test.

### Comparison of metabolic parameters of rats

**Figure 2.**
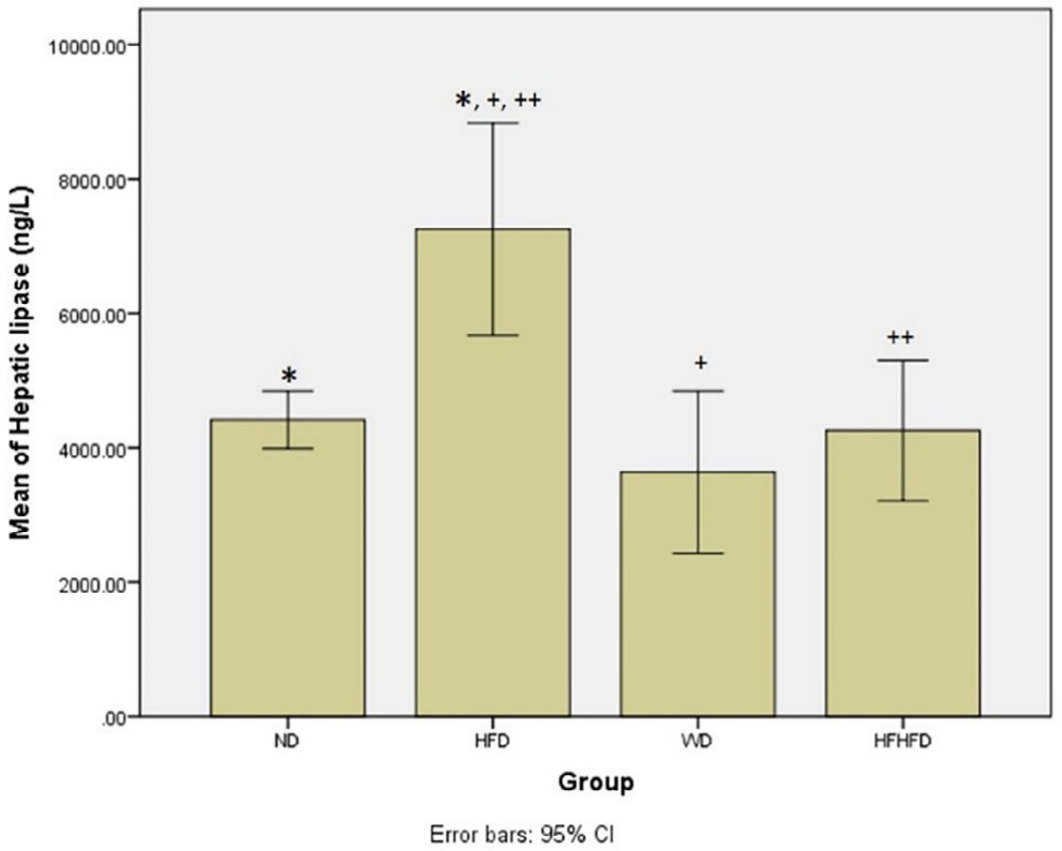
*Post hoc* analysis of hepatic lipase using Mann-Whitney Test. Note: Symbols represent significant post hoc result (p < 0.05). *p = 0.003 for ND vs HFD; +p = 0.003 for HFD vs WD; ++p = 0.015 for HFD vs HFHFD. ND: normal diet; HFD: high-fat diet; WD: western diet; HFHFD: high-fat-high-fructose diet.

Based on Table 1, regarding metabolic parameters, the highest average body weight and triglyceride levels were in the WD group, while the HFD group seemed to have the greatest increase in LDL. The HFD group had the highest levels of hepatic lipase, indicating a significant difference (p = 0.004) between the four groups. The *post hoc* test resulted in significant differences in hepatic lipase levels in the ND vs HFD, HFD vs WD, and HFD vs HFHFD groups ( Figure 2). From these results, the provision of fat-based diets affected the metabolic conditions of rats.

### Comparison of inflammatory and liver injury parameters of rats

**Figure 3.**
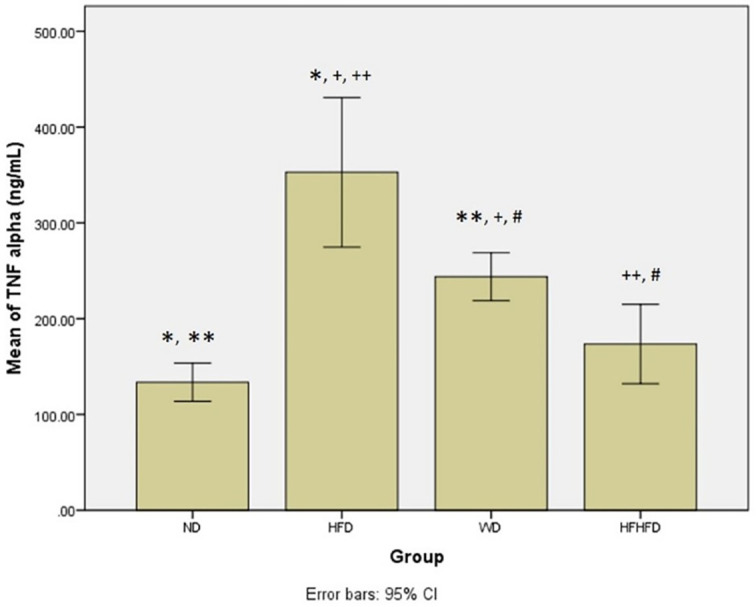
*Post hoc* analysis of TNF-α using Tukey HSD Test. Note: Symbols represent significant post hoc result (p < 0.05). *p = 0.000 for ND vs HFD; **p = 0.001 for ND vs WD; +p = 0.001 for HFD vs WD; ++p = 0.000 for HFD vs HFHFD; #p = 0.037 for WD vs HFHFD. ND: normal diet; HFD: high-fat diet; WD: western diet; HFHFD: high-fat-high-fructose diet.

**Figure 4.**
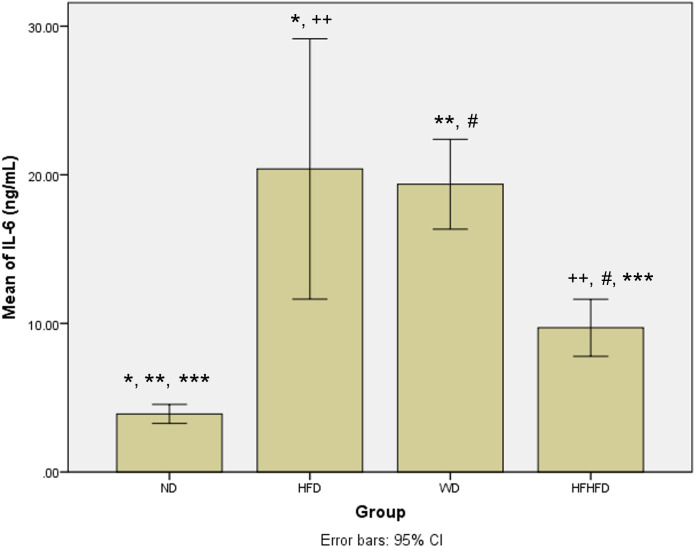
*Post hoc* analysis of IL-6 using Mann-Whitney Test. Note: Symbols represent significant *post hoc* result (p < 0.05). *p = 0.003 for ND vs HFD; **p = 0.002 for ND vs WD; ***p = 0.002 for ND vs HFHFD; ++p = 0.003 for HFD vs HFHFD; #p = 0.002 for WD vs HFHFD. ND: normal diet; HFD: high-fat diet; WD: western diet; HFHFD: high-fat-high-fructose diet.

**Figure 5.**
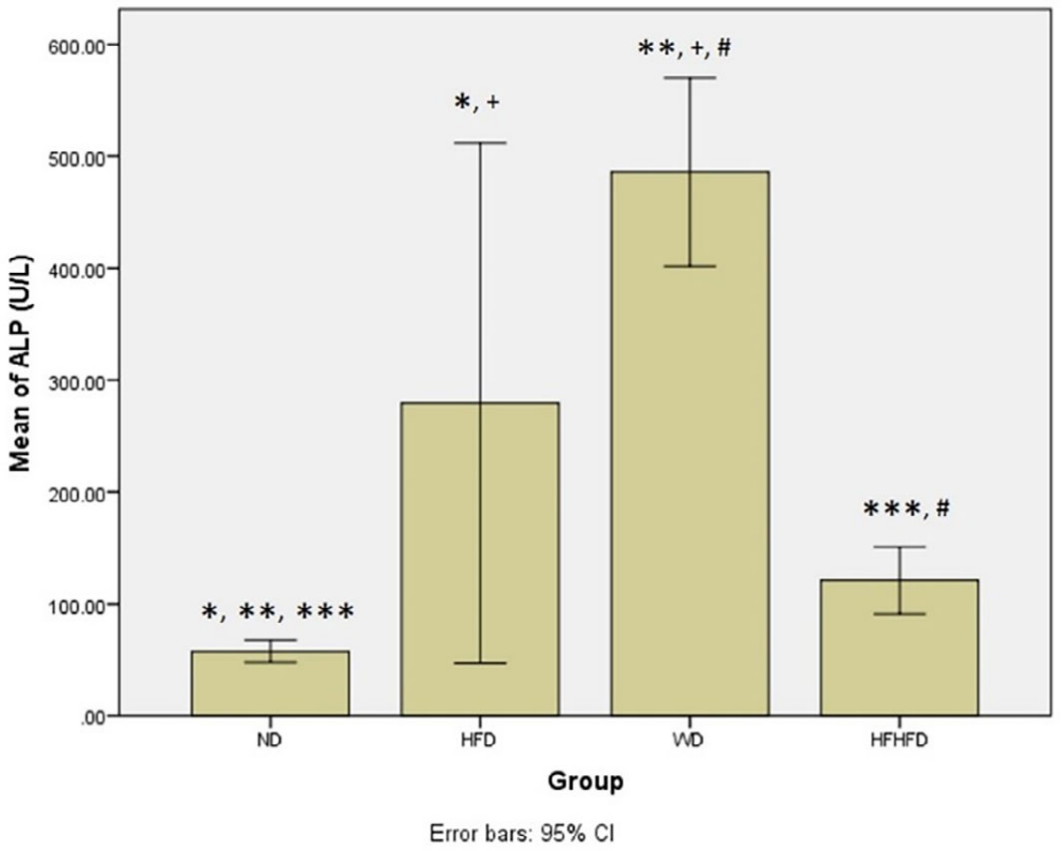
*Post hoc* analysis of ALP using Mann-Whitney Test. Note: Symbols represent significant post hoc result (p< 0.05). *p = 0.003 for ND vs HFD; **p = 0.002 for ND vs WD; ***p = 0.002 for ND vs HFHFD; +p = 0.046 for HFD vs WD; #p = 0.002 for WD vs HFHFD. ND: normal diet; HFD: high-fat diet; WD: western diet; HFHFD: high-fat-high-fructose diet.

When evaluating the inflammatory response and liver damage, TNF-α and IL-6, as well as liver enzymes like ALT, AST, ALP, and total bilirubin, are important parameters. ALT, AST, and total bilirubin did not show any significant differences between the four groups, as shown in Table 1. However, the levels of TNF-α and IL-6 were significantly different, and showed the highest levels in the HFD group, followed by WD, then HFHFD. The *post hoc* TNF-α test showed significant differences in all comparisons between groups, except for ND compared with HFHFD (p = 0.369) ( Figure 3). Meanwhile, in *post hoc* IL-6, the results were not significant only for HFD compared with WD (p = 0.568) ( Figure 4). The four groups also had significantly different levels of ALP, with WD having the highest levels, followed by HFD, then HFHFD ( Table 1). Based on the *post hoc* ALP test, the results were not significant only in the HFD group compared with the WD group ( Figure 5).

### Comparison of microbial dysbiosis of rats

**Figure 6.**
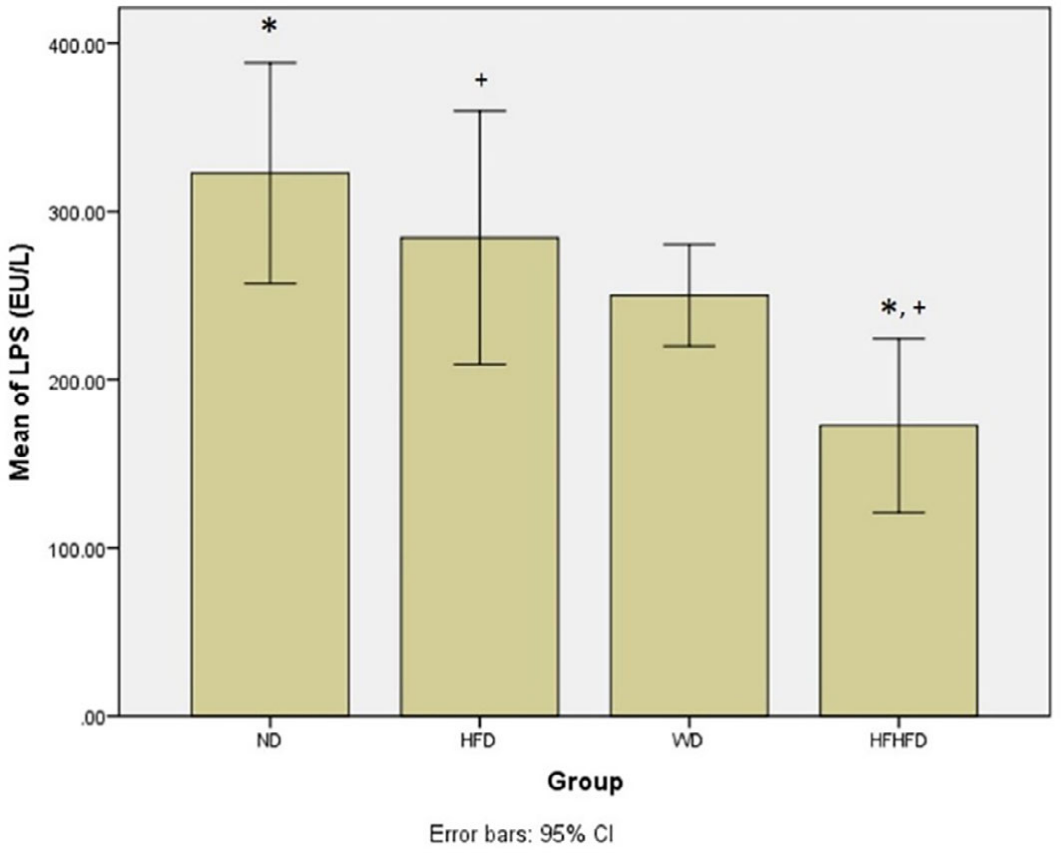
*Post hoc* analysis of LPS using Tukey HSD Test. Note: Symbols represent significant post hoc result (p< 0.05). *p = 0.001 for ND vs HFHFD; +p = 0.013 for HFD vs HFHFD. ND: normal diet; HFD: high-fat diet; WD: western diet; HFHFD: high-fat-high-fructose diet.

**Figure 7.**
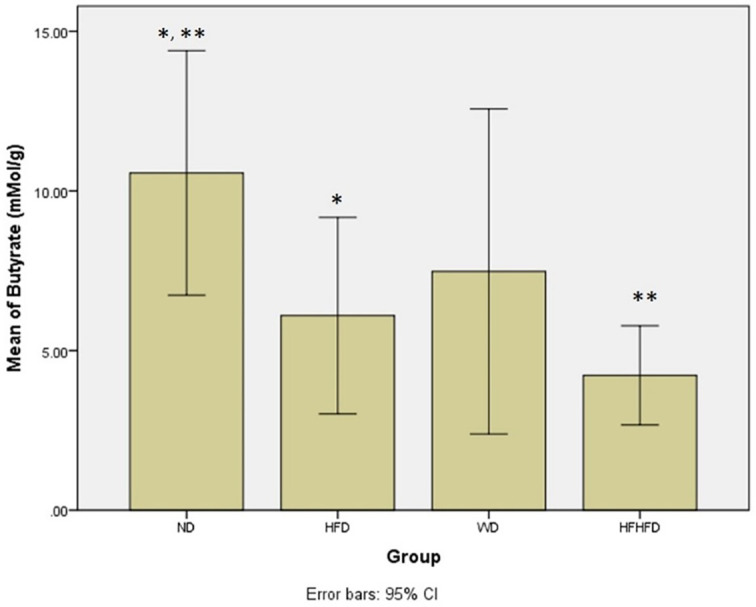
*Post hoc* analysis of butyrate using Mann-Whitney Test. Note: Symbols represent significant post hoc result (p< 0.05). *p = 0.046 for ND vs HFD; **p = 0.004 for ND vs HFHFD. ND: normal diet; HFD: high-fat diet; WD: western diet; HFHFD: high-fat-high-fructose diet.

Microbial dysbiosis is described by the parameters of LPS and SCFA levels. Based on Table 1, the LPS and butyrate levels were significantly different in all groups. The ND group had the highest LPS level, while the HFHFD group had the lowest. Based on LPS *post hoc* analysis ( Figure 6), there was a significant difference between ND vs HFHFD, and HFD vs HFHFD. The highest butyrate was in the ND group and the lowest was in the HFHFD. In *post hoc* analysis of butyrate ( Figure 7), we found p < 0.05 for ND vs HFHFD, and ND vs HFD.

### Comparison of liver histology of rats

**Figure 8.**
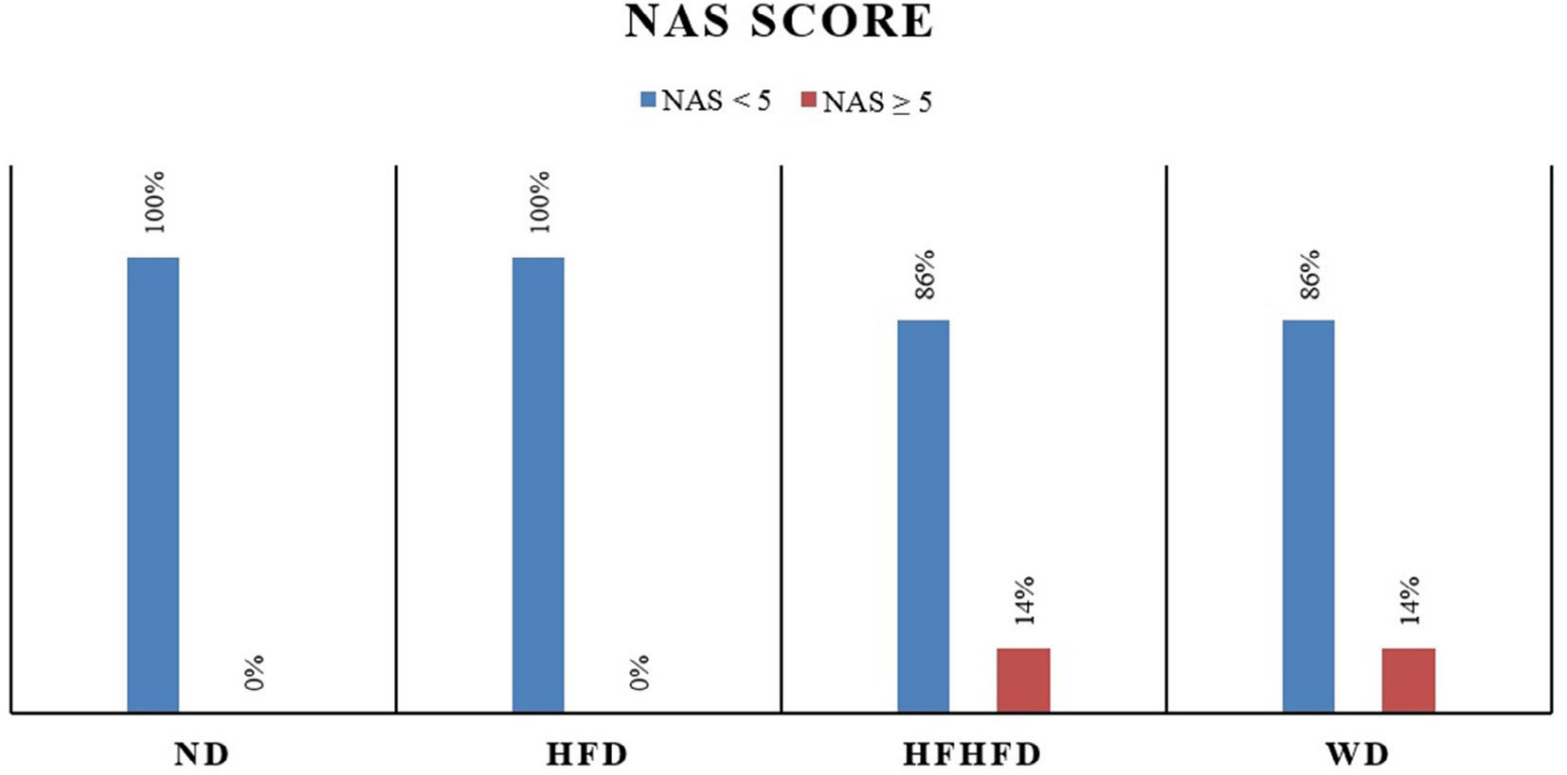
NAS percentages.

**Figure 9.**
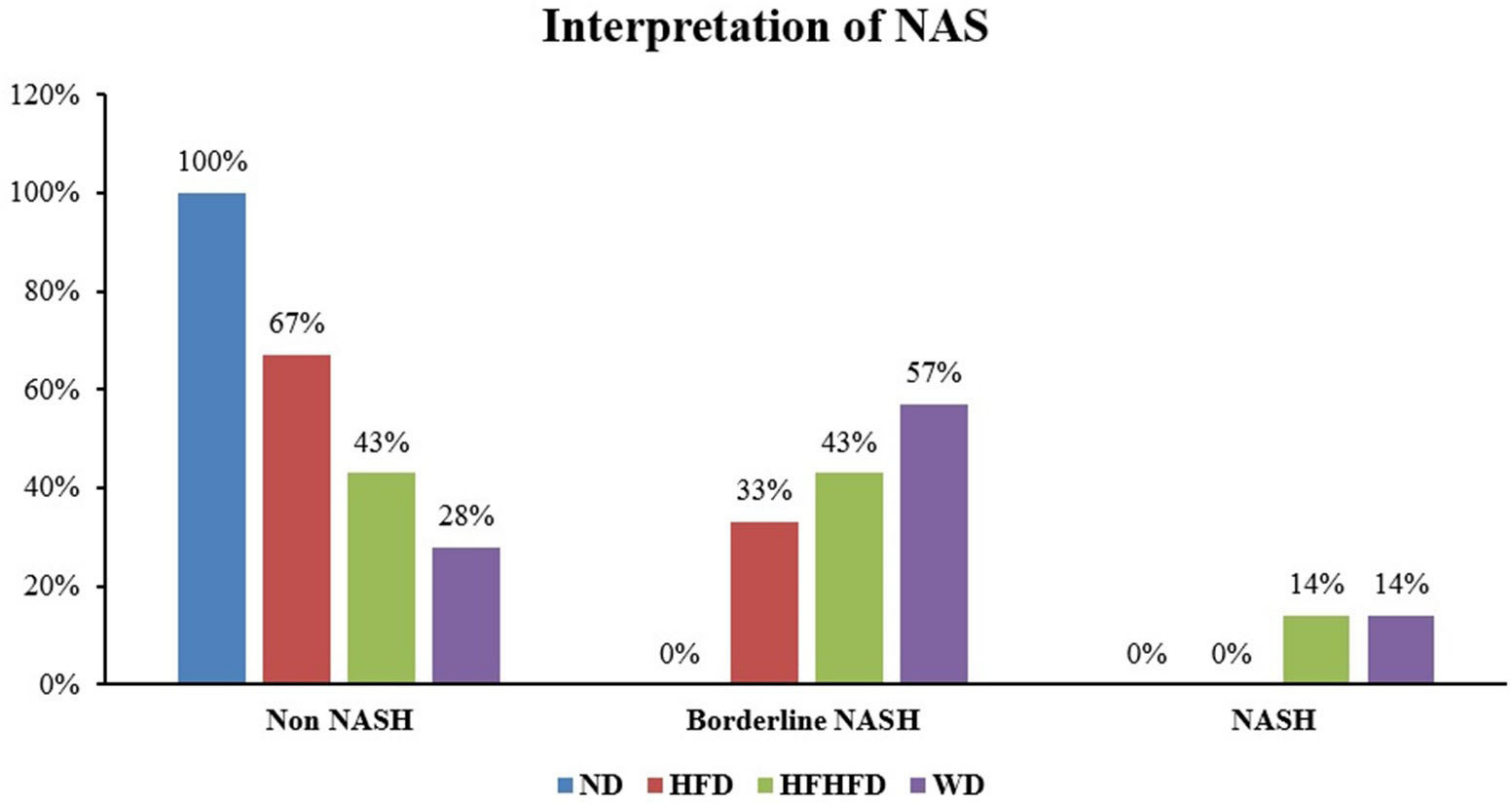
Interpretation of NAS.

**Figure 10.**
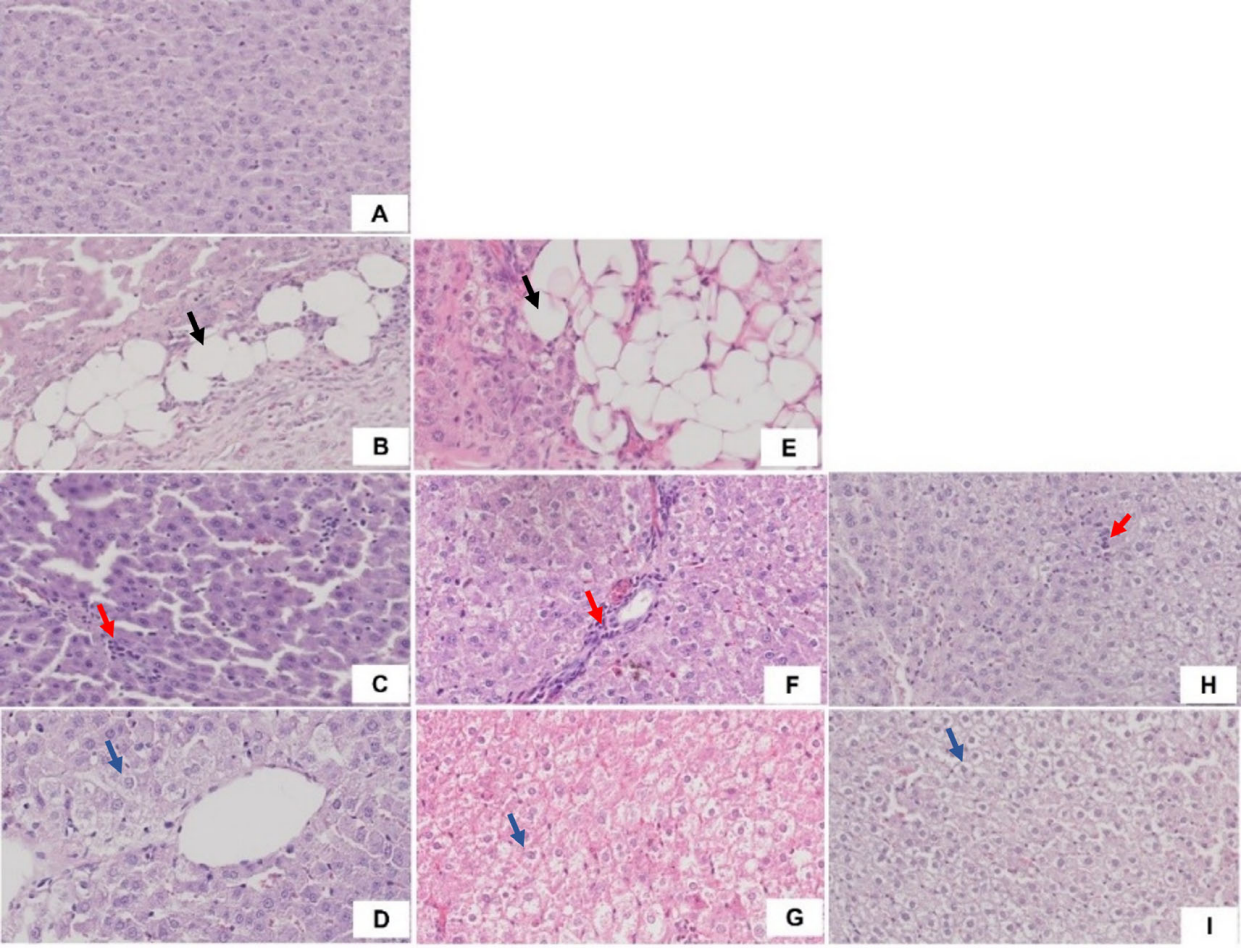
Liver histology. A). ND group; B). Steatosis in HFD group (black arrow); C). Lobular inflammation in HFD group (red arrow); D). Hepatocyte ballooning in HFD group (blue arrow); E). Steatosis in WD group (black arrow); F). Lobular inflammation in WD group (red arrow); G). Hepatocyte ballooning in WD group (blue arrow); H). Lobular inflammation in HFHFD group (red arrow); I). Hepatocyte ballooning in HFHFD group (blue arrow).

Liver histological analysis can be seen in Figure 10. Based on Table 2, the highest percentage of histological features in the HFD group was lobular inflammation; only 33.33% developed hepatocyte ballooning. Meanwhile, in the WD and HFHFD groups, all rats had lobular inflammation and most developed hepatocyte ballooning. Based on the NAS score, only the WD and HFHFD groups had NAS ≥5 with the same percentage ( Figure 8). The WD group had 57% of rats potentially experiencing NASH (borderline NAS), which was higher than the HFHFD group (43%) ( Figure 9).

**Table 2.** Liver histological findings.

Group	Steatosis	Lobular inflammation	Hepatocyte ballooning
ND (n = 7)	0%	0%	0%
HFD (n = 6)	16.67%	83.33%	33.33%
WD (n = 7)	14.28%	100%	85.71%
HFHD (n = 7)	0 %	100%	71.42%

## Discussion

Several factors have a role in the development of NASH, such as genetic variation, abnormal fat metabolism, oxidative stress, mitochondrial dysfunction, inflammatory response, and dysbiosis of gut microbiota. ^15^ Diet contributes to the pathophysiology of NAFLD. Some dietary consumption habits such as a high-fat and high-fructose diet can lead to liver fat accumulation and an increased risk of insulin resistance. ^16^ Boland *et al.* stated that a diet high in saturated fat and fructose plays a role in increasing oxidative stress and lipogenesis, stimulating an inflammation response, and triggering changes in the gut microbiota composition. ^17^ Different diet compositions can alter the natural course of NAFLD, therefore it is important to discuss the impact of different types of diet on the development of NAFLD.

Systemic low-grade inflammation, which has the potential to increase reactive oxygen species (ROS) and pro-oxidative stressors, is a hallmark of obesity. Obesity is associated with hyperglycemia and increased levels of free fatty acids (FFAs), which then induce lipotoxicity. ^18^ Increased FFAs and insulin resistance trigger hepatic steatosis. This condition has an impact on increasing hepatic lipase activity. Hydrolyzing hepatic triglycerides and lipoprotein phospholipids is the job of hepatic lipase. The degree of hepatic steatosis is positively correlated with hepatic lipase. ^19^ In our study, metabolic changes were found in the HFD, WD, and HFHFD groups. However, only rats on HFD and WD developed an obese phenotype by the end of the study, although the data were not significant. Triglyceride was found in the highest level in the WD group, while LDL was the highest in the HFD group. Hepatic lipase was found in excessive levels in the HFD group. A previous study proved that high-fat animals had significantly higher body weight than high-fructose animals. ^20^ Lee *et al.* stated that rats induced by high-fat and high-fat-high-fructose had significant higher body weights than high-fructose only. ^21^

The adiposity index, an increase in body weight, and excessive fat accumulation are all signs of obesity. Fat and sugar composition in the WD may contribute to increase of body weight by increasing abdominal fat mass and adiponectin expression in adipose tissue. Micronutrient composition in the WD could be possible factor that could affect a rat’s body weight gain. These results were in line with Bortolin et *al.* who concluded that the WD was the most effective diet to promote obesity in rats. Micronutrient content and diet palatability are the factors that contribute to weight gain in rats. ^22^

Circulating inflammatory cytokines are primarily derived from adipose tissue. Through the inflammatory pathway, high levels of circulating inflammatory signals can cause insulin resistance and provide positive feedback that increases liver inflammation. By activating the c-Jun N-terminal kinase (JNK) and nuclear factor-kappa B (NF-κB) signaling pathways, obesity increases the production of pro-inflammatory cytokines like TNF-α and IL-6. ^23^ In our study, obese rats that were in the HFD and WD groups also developed higher levels of TNF-α and IL-6. These results were also consistent with other studies that revealed the effect of HFD rat and obese diabetic patients on TNF-α and IL-6. ^24^^,^^25^

Cholesterol and saturated fatty acids (SFAs) are examples of WD ingredients that relate to the inflammatory response in the immune system. ^26^ Insulin resistance in the WD model may cause hypertriglyceridemia and hypercholesterolemia, which induce lipotoxicity and hepatic steatosis. SFAs and cholesterol accumulation in the WD could cause hepatic oxidative stress by disruption of the glutathione system and superoxide dismutase (SOD) levels. Furthermore, oxidative stress may trigger the activation of NF-κB, mitogen-activated protein kinase (MAPK), and the JNK cascade, which results in the increase of several cytokines such as TNF-α and IL-6 in the hepatocytes and Kupffer cells. ^27^

Metabolic changes and inflammatory conditions are closely related to the disruption of the intestinal barrier, leading to microbial dysbiosis. Gram-negative bacteria contain LPS, which in large quantities can induce an inflammatory response, leading to endotoxemia. The presence of LPS translocation that enters through the portal circulation can trigger the occurrence of repeated liver exposure, leading to liver injury. ^28^ Consuming high levels of fructose and fat was found to be strongly correlated with increased serum LPS levels, toll-like receptor 4 (TLR4) expression, as well as circulating cytokines. ^29^ A previous study confirmed the activation of the LPS–TLR4 pathway in obese rats induced by the HFHFD. ^30^ However, in our study, the results of LPS were not linear with other inflammatory cytokines (TNF-α and IL-6) and were inconsistent theoretically. This was caused by a short duration of intervention between groups.

Disruption of gut microbiota also contributes to the production of SCFA such as acetic, propionic, and butyric acids. Our study elucidated that the lower levels of butyric acid were found in HFHFD group (p = 0.03). Our findings were supported by those of previous studies. Consumption of HFHFD has previously been proven to affect the homeostasis of gut microbiota and increased cholesterol levels, which is associated with increased risk of intestinal disease such as Crohn’s disease, ulcerative colitis, and colon cancer. ^31^^,^^32^ Some supporting evidence also revealed that the levels of butyric acid in patients with ulcerative colitis and Crohn’s disease was lower than a healthy control, ^33^ indicating that butyric acid might have a protective effect against inflammatory bowel disease. In our study, a HFHFD might contribute to the disruption of gut microbiota homeostasis and thereafter cause the impaired production of butyric acid, a type of SCFA produced by gut microbiota in the colon. ^34^ This indicates that a HFHFD might cause a decrease of butyric acid levels.

The theory on how a HFHFD affects the levels of butyric acid remains to be properly defined. However, some previous studies have proposed a possible mechanism. Briefly, a HFHFD may cause the alteration of gut microbiota composition by reducing the *Megasphaera elsdenii* bacteria, a bacteria belonging to the *Firmicutes* group which have an ability to convert lactates into butyrate. In addition, after a HFHFD, it was reported that the beneficial *Bifidobacteria* and *Lactobacilli*, which interact with *Firmicutes* bacteria to produce butyric acid through cross-feeding, decreased in abundance. ^35^^,^^36^ This possible theory may explain the proposed mechanism on how a HFHFD affects the impaired production of butyric acid.

The duration, type of diet, and genetic factors all play a role in the development of NAFLD-associated liver histology. ^37^ In our study, steatosis, lobular inflammation, and hepatocyte ballooning were found in the HFD and WD groups, while only lobular inflammation and hepatocyte ballooning were found in the HFHFD group. NAS scores ≥ 5 were found in the WD and HFHFD groups. Although both the WD and HFHFD fulfill criteria of NASH histologically by the NAS score in the same percentage, we found a higher percentage of borderline NASH in the WD group. This result was in line with a previous study, which evaluated the effect of different diets (WD, cafeteria diet, and HFD) and found that the WD group had the highest steatosis score among others. ^22^

The development of NAFLD/NASH is influenced by metabolic factors, inflammatory factors, and microbiota dysbiosis factors that cause changes in liver histology. Research on the appropriate diet in inducing NAFLD/NASH needs to be analyzed based on these multifactors. For metabolic factors, administration of the HFD and WD causes obesity, while the WD and HFHFD cause an increase in triglycerides. This condition was related to the high levels of TNF-α and IL-6 in the three groups. Microbiota dysbiosis is characterized by impaired SCFA production, in this case found in the HFHFD group, which has the lowest butyrate levels among others. Based on histopathology, the WD and HFHFD groups met the criteria for the occurrence of NASH, but in percentage terms, borderline NASH was higher in the WD. We found that the WD cause changes in metabolic and inflammatory markers, but has less impact in SCFA production than HFHFD. However, it had more potential in liver histology alteration. We conclude that WD is the most appropriate diet-type model for NASH studies in rats.

The limitation of this study is that it did not assess how multiple types of high-fat diets affected different rat strains. In addition, further research regarding the different duration of food consumption needs to be compared and evaluated.

## Conclusions

In summary, different types of diet especially the WD significantly influenced inflammatory markers and dysbiosis for NASH progressivity in rats. The HFD group induced significant liver inflammation but did not produce NASH histologically, whereas the WD and HFHFD groups had the potential to develop NASH. So, among the four different types of diet, the WD is the most appropriate diet to induce NASH.

## Data Availability

Dryad. Data of Multiple Different High-Fat Diets. https://doi.org/10.5061/dryad.np5hqbzxx. ^38^ This project contains the following underlying data:
•
**Data file 1: Data of Normal Diet Conditioning**
Data files contain all measurements conducted during the ND conditioning of rats, including body weight, biochemical analysis using blood samples, SCFA analysis using feces, NAS analysis through liver histology, mean, Q1, Q3, and deviation standard of each measurement. •
**Data file 2: Data of High Fat Diet Conditioning**
Measurements conducted during the HFD conditioning of rats, including body weight, biochemical analysis using blood samples, SCFA using feces, NAS analysis through liver histology, mean, Q1, Q3, and deviation standard of each measurement. •
**Data file 3: Data of Western Diet Conditioning**
Measurements conducted during the WD conditioning of rats, including body weight, biochemical analysis using blood samples, SCFA analysis using feces, NAS analysis through liver histology, mean, Q1, Q3, and deviation standard of each measurement. •
**Data file 4: Data of High Fat High Fructose Diet Conditioning**
Measurements conducted during the HFHFD conditioning of rats, including the body weight, biochemical analysis using blood samples, SCFA analysis using feces, NAS analysis through liver histology, mean, Q1, Q3, and deviation standard of each measurement. •
**README.md**
README.md is a note that contains information and a summary of the dataset, as well as an explanation of the variables under study, the abbreviations, and units of measurement. •
**Related Work – Supplemental Information**
This project consists of the 10 supplemental figures, the document of SCFA analysis using shimadzu, and the full ARRIVE author checklist. Data are available under the terms of the Creative Commons Attribution 4.0 International. https://doi.org/10.5281/zenodo.7583400. ^39^ Data are available under the terms of the Dryad’s Term of Service and under the terms of the Creative Commons Zero “No rights reserved” data waiver (CC0 1.0 Public domain dedication).

## References

[1] Mitra S De A Chowdhury A : Epidemiology of non-alcoholic and alcoholic fatty liver diseases. *Transl Gastroenterol Hepatol.* 2020;5(16):1–17. 10.21037/tgh.2019.09.08 32258520 PMC7063528

[2] Omagari K Suzuta M Taniguchi A : A non-obese, diet-induced animal model of nonalcoholic steatohepatitis in Wistar/ST rats compared to Sprague-Dawley rats. *Clin Nutr Exp.* 2020;30:1–14. 10.1016/j.yclnex.2020.03.001

[3] Chalasani N Younossi Z Lavine JE : The diagnosis and management of nonalcoholic fatty liver disease: Practice guidance from the American Association for the Study of Liver Diseases. *Hepatology.* 2018;67(1):328–357. 10.1002/hep.29367 28714183

[4] Jarvis H Craig D Barker R : Metabolic risk factors and incident advanced liver disease in non-alcoholic fatty liver disease (NAFLD): A systematic review and meta-analysis of population-based observational studies. *PLoS Med.* 2020;17(4):e1003100. 10.1371/journal.pmed.1003100 32353039 PMC7192386

[5] Salehi A Sadat S Beigrezaei S : Dietary patterns and risk of non - alcoholic fatty liver disease. *BMC Gastroenterol.* 2021;21(41):1–12. 33509112 10.1186/s12876-021-01612-z PMC7844966

[6] Paik JM Henry L De Avila L : Mortality Related to Nonalcoholic Fatty Liver Disease Is Increasing in the United States. *Hepatol Commun.* 2019;3(11):1459–1471. 10.1002/hep4.1419 31701070 PMC6824058

[7] Ando Y Jou JH : Nonalcoholic Fatty Liver Disease and Recent Guideline Updates. *Clin. Liver Dis.* 2021;17(1):23–28. 10.1002/cld.1045 33552482 PMC7849298

[8] Handayani D Meyer BJ Chen J : A High-Dose Shiitake Mushroom Increases Hepatic Accumulation of Triacylglycerol in Rats Fed a High-Fat Diet: Underlying Mechanism. *Nutrients.* 2014;6:650–662. 10.3390/nu6020650 24566434 PMC3942724

[9] Stephenson K Kennedy L Hargrove L : Updates on Dietary Models of Nonalcoholic Fatty Liver Disease: Current Studies and Insights. *Gene Expr.* 2017;18(1):5–17. 29096730 10.3727/105221617X15093707969658 PMC5860971

[10] Linsenmeier RA Beckmann L Dmitriev AV : Intravenous ketamine for long term anesthesia in rats. *Heliyon.* 2020;6(12):e05686. 10.1016/j.heliyon.2020.e05686 33367124 PMC7749388

[11] Savari F Mard SA Badavi M : A new method to induce nonalcoholic steatohepatitis (NASH) in mice. *BMC Gastroenterol.* 2019;19(1):125. 10.1186/s12876-019-1041-x 31307427 PMC6632212

[12] Fan Y Xiong W Li J : Mechanism of TangGanJian on nonalcoholic fatty liver disease with type 2 diabetes mellitus. *Pharm Bio.* 2018;56(1):567–572. 10.1080/13880209.2018.1504972 30460863 PMC6249541

[13] Kamil RZ Murdiati A Juffrie M : Gut microbiota and short-chain fatty acid profile between normal and moderate malnutrition children in Yogyakarta, Indonesia. *Microorganisms.* 2021;9(1):1–15. 10.3390/microorganisms9010127 PMC7826765 33430510

[14] Lee G You HJ Bajaj JS : Distinct signatures of gut microbiome and metabolites associated with significant fibrosis in non-obese NAFLD. *Nat Commun.* 2020;11(1):1–13. 33020474 10.1038/s41467-020-18754-5 PMC7536225

[15] Marchisello S Di PA Scicali R : Pathophysiological, Molecular and Therapeutic Issues of Nonalcoholic Fatty Liver Disease: An Overview. *Int J Mol Sci.* 2019; (20,1948):1–33. 10.3390/ijms20081948 PMC6514656 31010049

[16] Buzzetti E Pinzani M Tsochatzis EA : The multiple-hit pathogenesis of non-alcoholic fatty liver disease (NAFLD). *Metabolism.* 2016;65(8):1038–1048. 10.1016/j.metabol.2015.12.012 26823198

[17] Boland ML Oró D Tølbøl KS : Towards a standard diet-induced and biopsy-confirmed mouse model of non-alcoholic steatohepatitis: Impact of dietary fat source. *World J Gastroenterol.* 2019;25(33):4904–4920. 10.3748/wjg.v25.i33.4904 31543682 PMC6737317

[18] Duan Y Zeng L Zheng C : Inflammatory Links Between High Fat Diets and Diseases. *Front Immunol.* 2018;9(2649):1–10. 30483273 10.3389/fimmu.2018.02649 PMC6243058

[19] Cedo L Santos D Rivas-urbina A : Human hepatic lipase overexpression in mice induces hepatic steatosis and obesity through promoting hepatic lipogenesis and white adipose tissue lipolysis and fatty acid uptake. *PLoS One.* 2017;12(12):1–14. 10.1371/journal.pone.0189834 PMC5731695 29244870

[20] Woodie L Blythe S : The differential effects of high-fat and high-fructose diets on physiology and behavior in male rats. *Nutr Neurosci.* 2017;21(5):328–336. 10.1080/1028415X.2017.1287834 28195006

[21] Lee JS Jun DW Kim EK : Histologic and metabolic derangement in high-fat, high-fructose, and combination diet animal models. *Sci World J.* 2015;2015(306326):1–9. 10.1155/2015/306326 26090514 PMC4451512

[22] Bortolin RC Vargas AR Gasparotto J : A new animal diet based on human Western diet is a robust diet-induced obesity model: Comparison to high-fat and cafeteria diets in term of metabolic and gut microbiota disruption. *Int J Obes.* 2018;42(3):525–534. 10.1038/ijo.2017.225 28895587

[23] Chen Z Yu R Xiong Y : A vicious circle between insulin resistance and inflammation in nonalcoholic fatty liver disease. *Lipids Health Dis.* 2017;16(1):1–9. 10.1186/s12944-017-0572-9 29037210 PMC5644081

[24] Goyal R Faizy AF Siddiqui SS : Evaluation of TNF-α and IL-6 Levels in Obese and Non-obese Diabetics: Pre- and Postinsulin Effects. *N Am J Med Sci.* 2012;4(4):180–184. 10.4103/1947-2714.94944 22536561 PMC3334258

[25] Adegbola PI Fadahunsi OS Ajilore BS : Combined ginger and garlic extract improves serum lipid profile, oxidative stress markers and reduced IL-6 in diet induced obese rats. *Obes Med.* 2021;23:100336. 10.1016/j.obmed.2021.100336

[26] Christ A Lauterbach M Latz E : Western Diet and the Immune System: An Inflammatory Connection. *Immunity.* 2019;51(5):794–811. 10.1016/j.immuni.2019.09.020 31747581

[27] Sabir U Muhammad H Ullah A : Downregulation of hepatic fat accumulation, inflammation and fibrosis by nerolidol in purpose built western-diet-induced multiple-hit pathogenesis of NASH animal model. *Biomed Pharmacother.* 2022;150(112956):112956. 10.1016/j.biopha.2022.112956 35447548

[28] An L Wirth U Koch D : The Role of Gut-Derived Lipopolysaccharides and the Intestinal Barrier in Fatty Liver Diseases. *J Gastrointest Surg.* 2022;26(3):671–683. 10.1007/s11605-021-05188-7 34734369 PMC8926958

[29] Lambertz J Weiskirchen S Landert S : Fructose: A dietary sugar in crosstalk with microbiota contributing to the development and progression of non-alcoholic liver disease. *Front Immunol.* 2017;8(1159). 10.3389/fimmu.2017.01159 28970836 PMC5609573

[30] Li KP Yuan M Wu YL : A High-Fat High-Fructose Diet Dysregulates the Homeostatic Crosstalk Between Gut Microbiome, Metabolome, and Immunity in an Experimental Model of Obesity. *Mol Nutr Food Res.* 2022;66(7):2100950. 10.1002/mnfr.202100950 35072983

[31] Hold GL Smith M Grange C : Role of the gut microbiota in inflammatory bowel disease pathogenesis: What have we learnt in the past 10 years? *World J Gastroenterol.* 2014;20(5):1192–1210. 10.3748/wjg.v20.i5.1192 24574795 PMC3921503

[32] Sánchez-Alcoholado L Ramos-Molina B Otero A : The role of the gut microbiome in colorectal cancer development and therapy response. *Cancers (Basel).* 2020;12(1406):1–29. 10.3390/cancers12061406 PMC7352899 32486066

[33] Tang X Li X Wang Y : Butyric Acid Increases the Therapeutic Effect of EHLJ7 on Ulcerative Colitis by Inhibiting JAK2/ STAT3/SOCS1 Signaling Pathway. *Front Pharmacol.* 2020;10(1553):1–10. 10.3389/fphar.2019.01553 PMC6987075 32038241

[34] Onyszkiewicz M Gawrys-kopczynska M Konopelski P : Butyric acid, a gut bacteria metabolite, lowers arterial blood pressure via colon-vagus nerve signaling and GPR41/43 receptors. *Pflugers Arch.* 2019;471:1441–1453. 10.1007/s00424-019-02322-y 31728701 PMC6882756

[35] Horne RG Yu Y Zhang R : High Fat-High Fructose Diet-Induced Changes in the Gut Microbiota Associated with Dyslipidemia in. *Nutrients.* 2020;12(11):3557. 10.3390/nu12113557 33233570 PMC7699731

[36] Markowiak-kope P Śliżewska K : The Effect of Probiotics on the Production of Short-Chain Fatty Acids by Human Intestinal Microbiome. *Nutrients.* 2020;12(4):1107. 10.3390/nu12041107 32316181 PMC7230973

[37] Kucera O Cervinkova Z : Experimental models of non-alcoholic fatty liver disease in rats. *World J Gastroenterol.* 2014;20(26):8364–8376. 10.3748/wjg.v20.i26.8364 25024595 PMC4093690

[38] Mustika S Santosaningsih D Handayani D : Data of Multiple Different High-Fat Diets. *Dryad.* 2023. 10.5061/dryad.np5hqbzxx PMC11369591 39229607

[39] Mustika S Santosaningsih D Handayani D : Data of multiple different high-fat diets. *Zenodo.* 2023. 10.5281/zenodo.7583400 PMC11369591 39229607

