# Impact of multiple different high-fat diets on metabolism, inflammatory markers, dysbiosis, and liver histology: study on NASH rat model induced diet

**DOI:** 10.12688/f1000research.129645.2

**Published:** 2023-12-12

**Authors:** Syifa Mustika, Dewi Santosaningsih, Dian Handayani, Achmad Rudijanto

**Affiliations:** 1 Doctoral Program of Medical Science, Faculty of Medicine, Universitas Brawijaya, Malang, Jl. Veteran, 65145, Indonesia; 2 Department of Clinical Microbiology, Faculty of Medicine, Universitas Brawijaya, Malang, Jl. Veteran, 65145, Indonesia; 3 Department of Nutrition, Faculty of Health Science, Universitas Brawijaya, Malang, Jl. Veteran, 65145, Indonesia; 4 Endocrine Metabolic & Diabetes Division, Department of Internal Medicine, Faculty of Medicine, Universitas Brawijaya - Dr Saiful Anwar Hospital, Malang, Jl. Veteran, 65145, Indonesia

**Keywords:** diet, non-alcoholic steatohepatitis, Rattus norvegicus strain Wistar

## Abstract

**Background:**

The spectrum of non-alcoholic fatty liver disease (NAFLD), known as non-alcoholic steatohepatitis (NASH), can lead to advanced liver disease. It is known that a variety of diets play a significant role in the development of NAFLD/NASH. The goal of this study was to determine the most appropriate composition of diet to induce NASH in an animal model.

**Methods:**

This research used *Rattus norvegicus* strain Wistar (n=27), which were divided into four groups and given each diet for 12 weeks: normal diet (ND, n=7), high-fat diet (HFD, n=6), western diet (WD, n=7), and high-fat-high-fructose diet (HFHFD, n=7). Subjects were monitored for changes in body weight. Blood samples were collected for biochemical analysis, including *low-density lipoprotein* (LDL), triglyceride, alanine aminotransferase (ALT), aspartate aminotransferase (AST), alkaline phosphatase (ALP), hepatic lipase, tumor necrosis factor-alpha (TNF-α), interleukin-6 (IL-6), and lipopolysaccharide (LPS). Fecal samples were taken for *short-chain fatty acid* (SCFA) analysis. Liver histology was assessed using NAS (NAFLD activity score). A statistical comparison test was carried out using the one-way ANOVA or Kruskal–Wallis test.

**Results:**

The highest average body weight was observed in the WD group (346.14 g). Liver enzymes, LDL, triglyceride, propionic acid, and acetic acid did not show significantly differences among the groups. TNF-α, IL-6, and hepatic lipase were significant (p = 0.000; p = 0.000; p = 0.004) and the highest level recorded in the HFD group. Butyrate acid level also showed significances (p = 0.021) with the lowest concentration seen in the HFHFD group (4.77 mMol/g). Only WD and HFHFD had a NAS ≥ 5 (14% and 14%). The highest percentage of borderline NAS was found in WD (57%).

**Conclusions:**

WD feeding is the most appropriate diet type to induce NASH in rats as it influences metabolic, inflammatory, dysbiosis, and liver histology of rats.

## Introduction

Non-alcoholic fatty liver disease (NAFLD) is becoming a common medical problem due to of its high incidence and treatment complexity. According to the most recent epidemiology data NAFLD has become the second most common liver disease after viral hepatitis, with an incidence rate of 20–30%, and obesity affecting up to 57.74% in the global population. ^1^

The subtype of NAFLD, known as non-alcoholic steatohepatitis (NASH), has emerged as a significant public health concern. ^2^ NASH is defined via liver biopsy as the presence of ≥5% hepatic steatosis and inflammation, accompanied by hepatocyte injury ( *e.g.,* ballooning), with or without any fibrosis. It is a potentially progressive liver disease that can lead to cirrhosis. ^3^ Risk factors for the development of NASH include excessive calorie-dense food intake, lack of physical activity and exercise, and genetic susceptibility. ^4^

Poor dietary habits may induce NASH, directly by affecting hepatic triglyceride accumulation and antioxidant activity, and indirectly by impairing insulin sensitivity and fat metabolism. ^5^ According to a previous study, the total prevalence of NAFLD is expected to increase by 33.5% by 2030. This condition is associated with a significantly increased incidence of NASH complications, such as decompensated cirrhosis (168%), hepatocellular carcinoma (137%), and liver-related mortality (178%). ^6^

Clinical experiment using humans as research subjects in NAFLD/NASH is limited by ethical considerations, as it involves liver biopsy as the gold standard for NAFLD/NASH. In addition, the development of NASH in humans can take a long time, up to several decades. ^7^ Therefore, research related to NASH requires experimental animal models and appropriate exposure to represent the biology and clinical outcomes of NASH as in humans. The lack of preclinical models that mimic human NASH poses obstacles to the elucidation of disease mechanisms and drug development.

The type of diet is an important factor in the development and progression of various metabolic diseases. Various high-fat diets have been used to induce NASH in experimental animals. The high-fat diet (HFD), western diet (WD), and high-fat-high-fructose diet (HFHFD) are the types of diet used to induce NASH. ^8^^,^^9^ Although previous studies used various diets, there are still no established dietary standards for creating experimental animal models of NASH.

This study aims to compare and determine the most representative diet for inducing NASH in the *Rattus norvegicus* Wistar strain. To ascertain whether the rats have developed NASH, many parameters including metabolic, inflammatory, and liver damage, microbial dysbiosis, and liver histology, were investigated.

## Methods

### Animals and diet

The Ethical Committee of the Faculty of Medicine, Universitas Brawijaya reviewed and approved all procedures (No. 66/EC/KEPK/02/2021). A total of 27 male Wistar rats were obtained from Universitas Gadjah Mada. Rat inclusion criteria included: male rat with shiny white fur, healthy, active, and had normal behavior; about 8-12 weeks old; the average body weight was 150-180 grams. Exclusion criteria included: the appearance of dull fur, loss and baldness; less or inactive activity; rats that during the study did not want to eat; weight loss >10% after the adaptation period; disabled, sick, and/or dead rats. This research used the refinement principle to ensure the welfare of experimental animals until the end of the study to minimize pain and discomfort. Food and drink were provided regularly every day with a certain type of diet according to the type of treatment. Cage maintenance, cage cleaning, and wood husk replacement were carried out every day with attention to light, temperature, and humidity. Rats’ conditions were monitored and evaluated every day and placed them individually in each cage. Before being treated, the rats were acclimatized for two weeks, given a standard diet, and placed inside cages at the Pharmacology Laboratory, Faculty of Medicine, Universitas Brawijaya. The Wistar rats were randomly assigned using a table of random numbers, then categorized into four groups: normal diet (ND) (67% carbohydrate, 21% protein, 7% fat, 5% fiber); HFD (67.1% carbohydrate, 16.5% fat, 16.4% protein), WD (52% carbohydrate, 16.1% protein, 31.7% fat), and HFHFD (41.5% carbohydrate, 10.3% fat, 10.2% protein, 38% fructose). All diets were given for 12 weeks. After the last administration of diet intervention, the rats fasted for 12 hours but consumed water freely. After that, all groups were euthanized with ketamine–xylazine intravenously to relieve pain on the same day before surgery was performed. ^10^ The blood serum, fecal, and liver were taken for further testing.

### Biochemical measurements and assays

Rat serum was used to analyze biochemical parameters in the Clinical Pathology Laboratory, Universitas Brawijaya, Indonesia. ^11^ Serum alanine aminotransferase (ALT), aspartate aminotransferase (AST), and alkaline phosphatase (ALP) were chemically analyzed using colorimetric analysis (ADVIA 2400 Clinical Chemistry System (Siemens, Germany). Serum hepatic lipase, tumor necrosis factor-alpha (TNF-α), interleukin-6 (IL-6), and lipopolysaccharide (LPS) were analyzed using the sandwich enzyme-linked immunosorbent assay (ELISA) method. ^12^

### Fecal sample preparation and SCFA measurement

A total of 0.5 grams of fecal samples from the colon were collected, labeled, and placed into container tubes. These samples were immediately stored at −40°C until the analysis day. At the time of analysis, 0.2 grams of fecal sample supernatant was poured into a 2 mL microtube and then added with sterile aquabidest water for injection. This suspension underwent 20 minutes of sonification, followed by centrifugation (14,000 rpm, 4°C, 10 min). The second centrifugation step (1,000 rpm, 4°C, 10 min) was performed while the natant was discarded. The final supernatant was injected into a gas chromatography (Shimadzu, GC-2010 Plus, Kyoto, Japan). Fecal pH measurement was used using a pH meter (pH Spear Eutech, Eutech Instruments, Paisley, United Kingdom). ^13^ This procedure was performed at the Food Technology and Agricultural Products Laboratory, Universitas Gadjah Mada, Indonesia.

### Histopathology assessment of NAS

The liver was sliced, fixed with 10% buffered formalin, embedded in paraffin, and stained with hematoxylin–eosin (HE) stain at a thickness of 5 μm. ^11^ Sample preparation was conducted at the Anatomical Pathology Laboratory of Universitas Brawijaya, Indonesia. The NAFLD Activity Score (NAS) was used to assess liver histology. Three parameters (steatosis score 0–3; lobules inflammation score 0–3; ballooning score 0–2) were utilized to determine NAFLD staging. Scores of 0–2 are defined as non-NASH, scores of 3–4 are defined as borderline, while scores ≥ 5 are considered diagnostic of NASH. ^14^

### Statistical analysis

Data were presented as the mean ± standard deviation and were analyzed with SPSS 25.0 (RRID: SCR_002865) for Windows. A one-way ANOVA was conducted when the data were normally distributed and was followed by the Tukey Honest Significant Difference (HSD) *post hoc* test if the data were significant. The Kruskal–Wallis test was used when the data distribution was not normal. If the results were significant then the Mann-Whitney test was performed. A p-value of <0.05 was considered significant.

**Figure 1.**
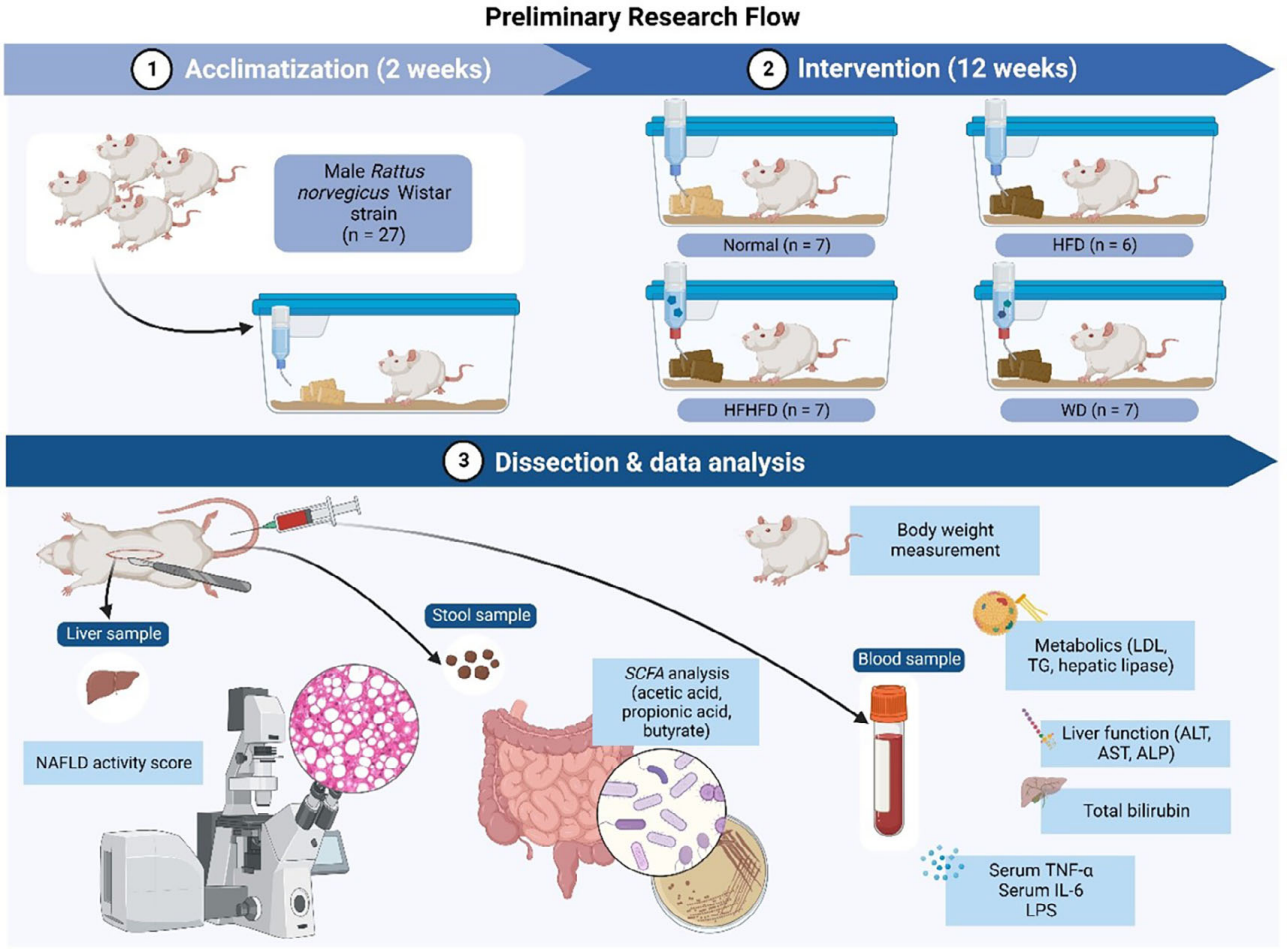
Research flow.

The research flow ( Figure 1) consisted of the following steps: 1). Acclimatization of Rattus norvegicus for two weeks; 2). Implementing various dietary interventions (ND, HFD, WD, HFHFD) for 12 weeks; 3). Dissection and data analysis at the end of the process. Various samples such as liver, stool, and blood were collected for further analysis.

## Results

This research was done by following the method and research flow that has been explained above. Comparison of various diets induction in rats after 12 weeks of intervention was performed as in Table 1.

**Table 1.** Comparison of various diets induction in rats after 12 weeks of intervention.

Parameters	ND (Mean ± SD)	HFD (Mean ± SD)	WD (Mean ± SD)	HFHFD (Mean ± SD)	p
Metabolic					
Body weight (g)	294.57 ± 8.73	326.67 ± 23.69	346.14 ± 55.60	285.43 ± 63.87	0.154 ^#^
LDL (mg/dL)	31.33 ± 9.35	35.17 ± 10.92	24.19 ± 7.48	20.25 ± 9.01	0.056 ^*^
Triglyceride (mg/dL)	79.71 ± 27.88	72.17 ± 33.58	125.14 ± 78.10	82.43 ± 39.70	0.354 ^#^
Hepatic lipase (ng/L)	4417.86 ± 430.54	7249.83 ± 1372.31	3637.86 ± 1210.17	4257.08 ± 1046.45	0.004 ^#^
Inflammatory and liver injury					
AST (U/L)	113.29 ± 25.20	104.50 ± 42.04	72.86 ± 15.30	88.14 ± 29.18	0.11 ^*^
ALT (U/L)	48.00 ± 12.01	57.00 ± 20.45	39.43 ± 12.23	44.57 ± 17.80	0.337 ^*^
Total bilirubin (mg/dL)	0.44 ± 0.07	0.48 ± 0.04	0.38 ± 0.08	0.47 ± 0.04	0.112 ^#^
TNF-α (ng/mL)	133.64 ± 20.01	352.88 ± 67.88	243.83 ± 25.07	173.57 ± 41.44	0.000 ^*^
IL-6 (ng/mL)	3.91 ± 0.64	20.39 ± 7.62	19.36 ± 3.03	9.70 ± 1.91	0.000 ^#^
ALP (U/L)	57.57 ± 9.76	279.50 ± 202.10	485.86 ± 84.26	120.86 ± 30.12	0.000 ^#^
Microbial dysbiosis					
LPS (EU/L)	322.70 ± 65.67	284.40 ± 65.55	250.06 ± 30.31	172.68 ± 51.71	0.001 ^*^
Acetic acid (mMol/g)	66.43 ± 7.27	61.85 ± 13.01	68.18 ± 23.82	54.60 ± 9.79	0.419 ^#^
Propionic acid (mMol/g)	21.28 ± 4.65	25.19 ± 5.06	29.69 ± 11.81	28.03 ± 8.16	0.316 ^#^
Butyric acid (mMol/g)	10.56 ± 3.83	6.10 ± 2.68	7.47 ± 5.09	4.77 ± 1.48	0.021 ^#^
Liver histology scoring					
NAS	-	2.16 ± 0.69	3.42 ± 1.29	2.85 ± 1.24	0.209 ^*^

^*^ One-way ANOVA test.

^#^ Kruskal–Wallis test.

### Comparison of metabolic parameters of rats

**Figure 2.**
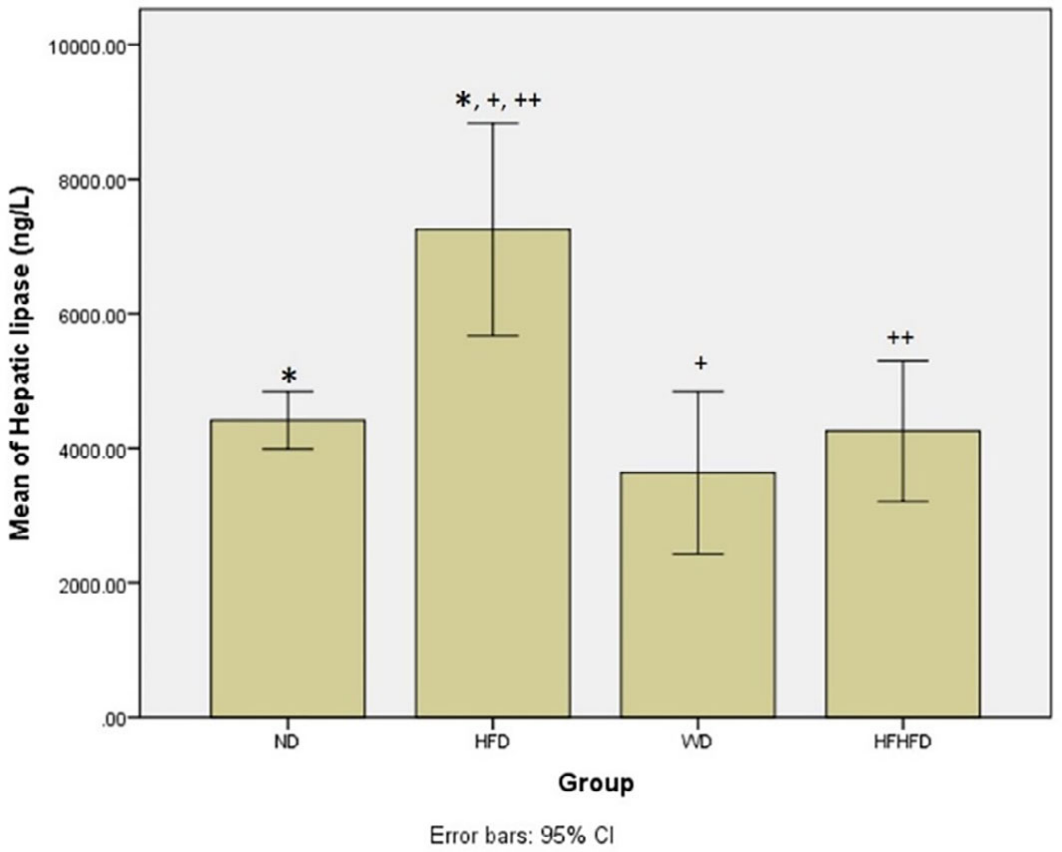
*Post hoc* analysis of hepatic lipase using Mann-Whitney Test. Note: Symbols represent significant post hoc result (p < 0.05). *p = 0.003 for ND vs HFD; +p = 0.003 for HFD vs WD; ++p = 0.015 for HFD vs HFHFD. ND: normal diet; HFD: high-fat diet; WD: western diet; HFHFD: high-fat-high-fructose diet.

Based on Table 1, regarding metabolic parameters, the highest average body weight and triglyceride levels were in the WD group, while the HFD group seemed to have the greatest increase in LDL. The HFD group had the highest levels of hepatic lipase, indicating a significant difference (p = 0.004) between the four groups. The *post hoc* test resulted in significant differences in hepatic lipase levels in the ND vs HFD, HFD vs WD, and HFD vs HFHFD groups ( Figure 2). From these results, the provision of fat-based diets affected the metabolic conditions of rats.

### Comparison of inflammatory and liver injury parameters of rats

**Figure 3.**
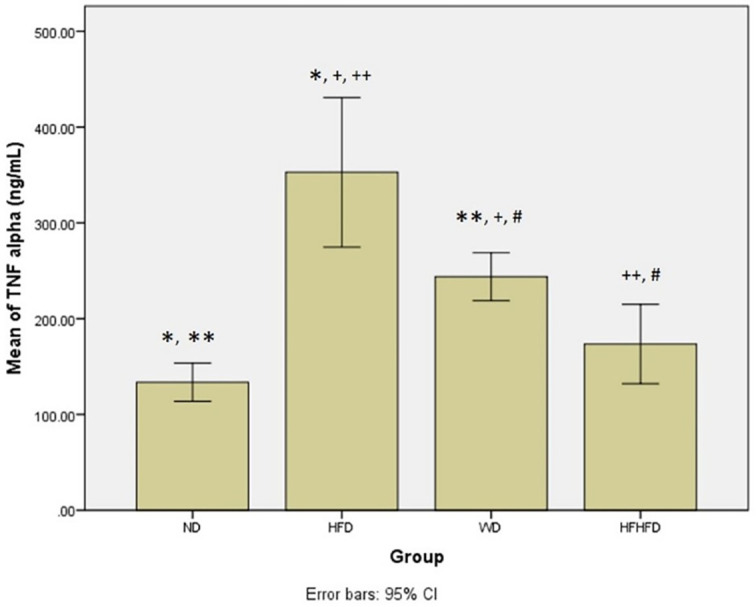
*Post hoc* analysis of TNF-α using Tukey HSD Test. Note: Symbols represent significant post hoc result (p < 0.05). *p = 0.000 for ND vs HFD; **p = 0.001 for ND vs WD; +p = 0.001 for HFD vs WD; ++p = 0.000 for HFD vs HFHFD; #p = 0.037 for WD vs HFHFD. ND: normal diet; HFD: high-fat diet; WD: western diet; HFHFD: high-fat-high-fructose diet.

**Figure 4.**
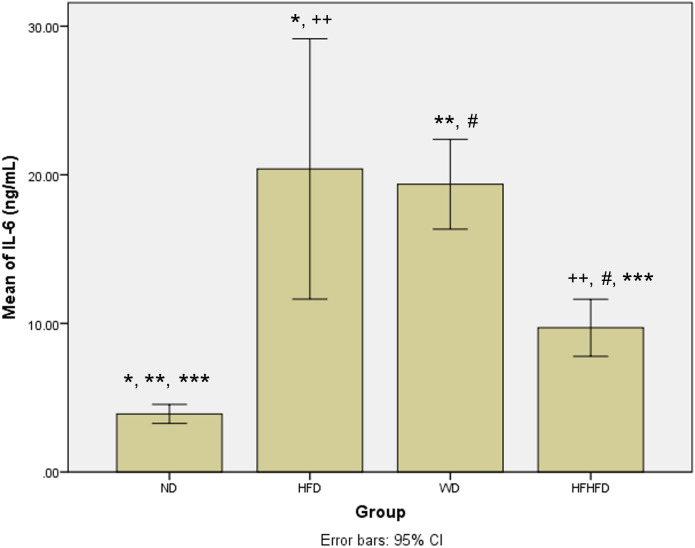
*Post hoc* analysis of IL-6 using Mann-Whitney Test. Note: Symbols represent significant *post hoc* result (p < 0.05). *p = 0.003 for ND vs HFD; **p = 0.002 for ND vs WD; ***p = 0.002 for ND vs HFHFD; ++p = 0.003 for HFD vs HFHFD; #p = 0.002 for WD vs HFHFD. ND: normal diet; HFD: high-fat diet; WD: western diet; HFHFD: high-fat-high-fructose diet.

**Figure 5.**
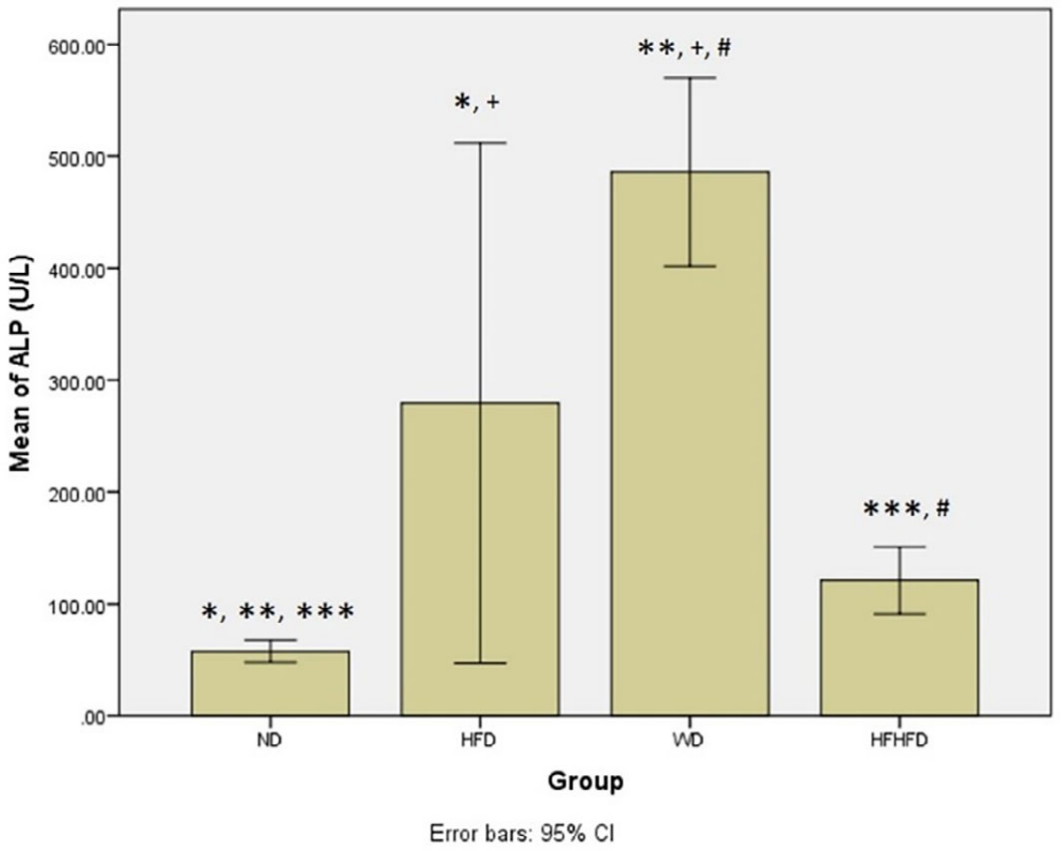
*Post hoc* analysis of ALP using Mann-Whitney Test. Note: Symbols represent significant post hoc result (p< 0.05). *p = 0.003 for ND vs HFD; **p = 0.002 for ND vs WD; ***p = 0.002 for ND vs HFHFD; +p = 0.046 for HFD vs WD; #p = 0.002 for WD vs HFHFD. ND: normal diet; HFD: high-fat diet; WD: western diet; HFHFD: high-fat-high-fructose diet.

When evaluating the inflammatory response and liver damage, parameters such as TNF-α and IL-6, as well as liver enzymes like ALT, AST, ALP, and total bilirubin, play a crucial role. ALT, AST, and total bilirubin did not exhibit any significant differences among the four groups, as shown in Table 1. However, the levels of TNF-α and IL-6 were significantly different, with the highest levels observed in the HFD group, followed by WD, and then HFHFD. The *post hoc* test TNF-α test revealed significant differences in all group comparisons between groups, except for ND compared to HFHFD (p = 0.369) ( Figure 3). Meanwhile, in the case of *post hoc* IL-6 analysis, the results were not significant only for HFD compared to WD (p = 0.568) ( Figure 4). Furthermore, all four groups had significantly different ALP levels, with WD having the highest levels, followed by HFD, then HFHFD ( Table 1). Based on the *post hoc* ALP test, the results were not significant only in the HFD group compared with the WD group ( Figure 5).

### Comparison of microbial dysbiosis of rats

**Figure 6.**
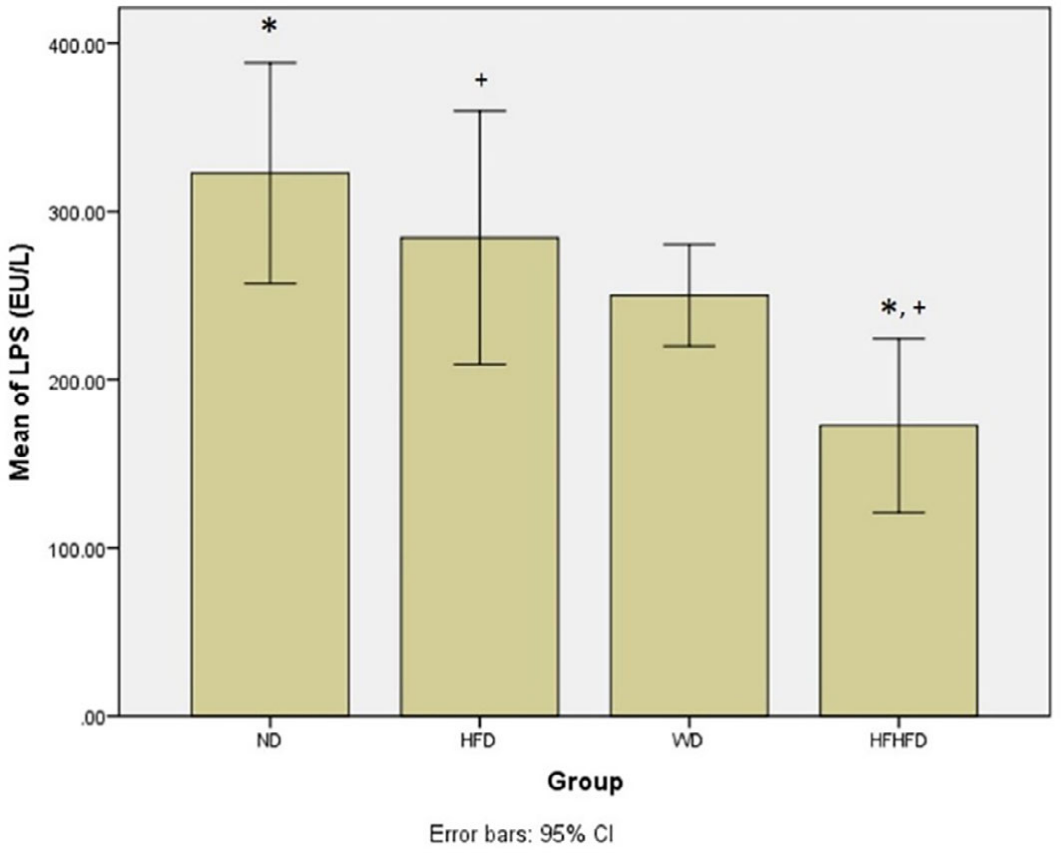
*Post hoc* analysis of LPS using Tukey HSD Test. Note: Symbols represent significant post hoc result (p < 0.05). *p = 0.001 for ND vs HFHFD; +p = 0.013 for HFD vs HFHFD. ND: normal diet; HFD: high-fat diet; WD: western diet; HFHFD: high-fat-high-fructose diet.

**Figure 7.**
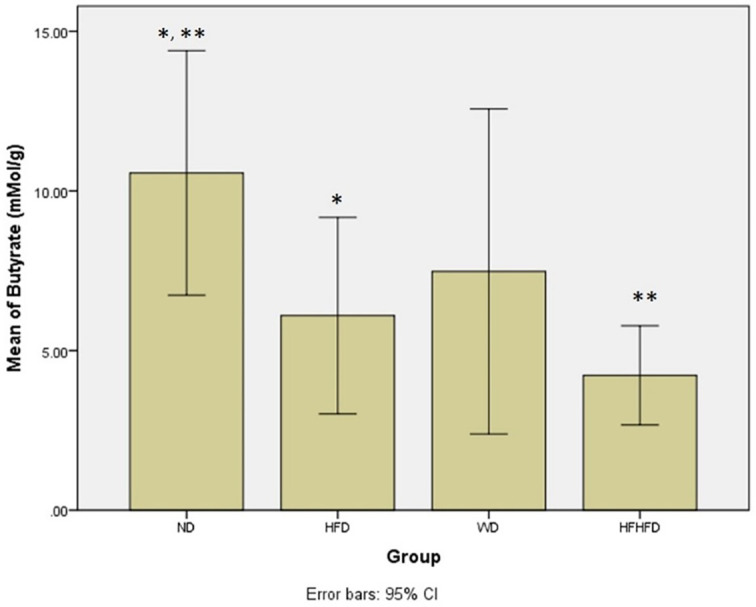
*Post hoc* analysis of butyric acid using Mann-Whitney Test. Note: Symbols represent significant post hoc result (p< 0.05). *p = 0.046 for ND vs HFD; **p = 0.004 for ND vs HFHFD. ND: normal diet; HFD: high-fat diet; WD: western diet; HFHFD: high-fat-high-fructose diet.

Microbial dysbiosis is described by the parameters of LPS and SCFA levels. According to Table 1, both LPS and butyric acid levels displayed significant differences across all groups. The ND group exhibited the highest LPS level, while the HFHFD group had the lowest. LPS *post hoc* analysis ( Figure 6), indicated significant differences between ND vs HFHFD, and HFD vs HFHFD. The highest butyric acid was in the ND group and the lowest was in the HFHFD. In *post hoc* analysis of butyric acid ( Figure 7), showed that p < 0.05 for ND vs HFHFD, and ND vs HFD.

### Comparison of liver histology of rats

**Figure 8.**
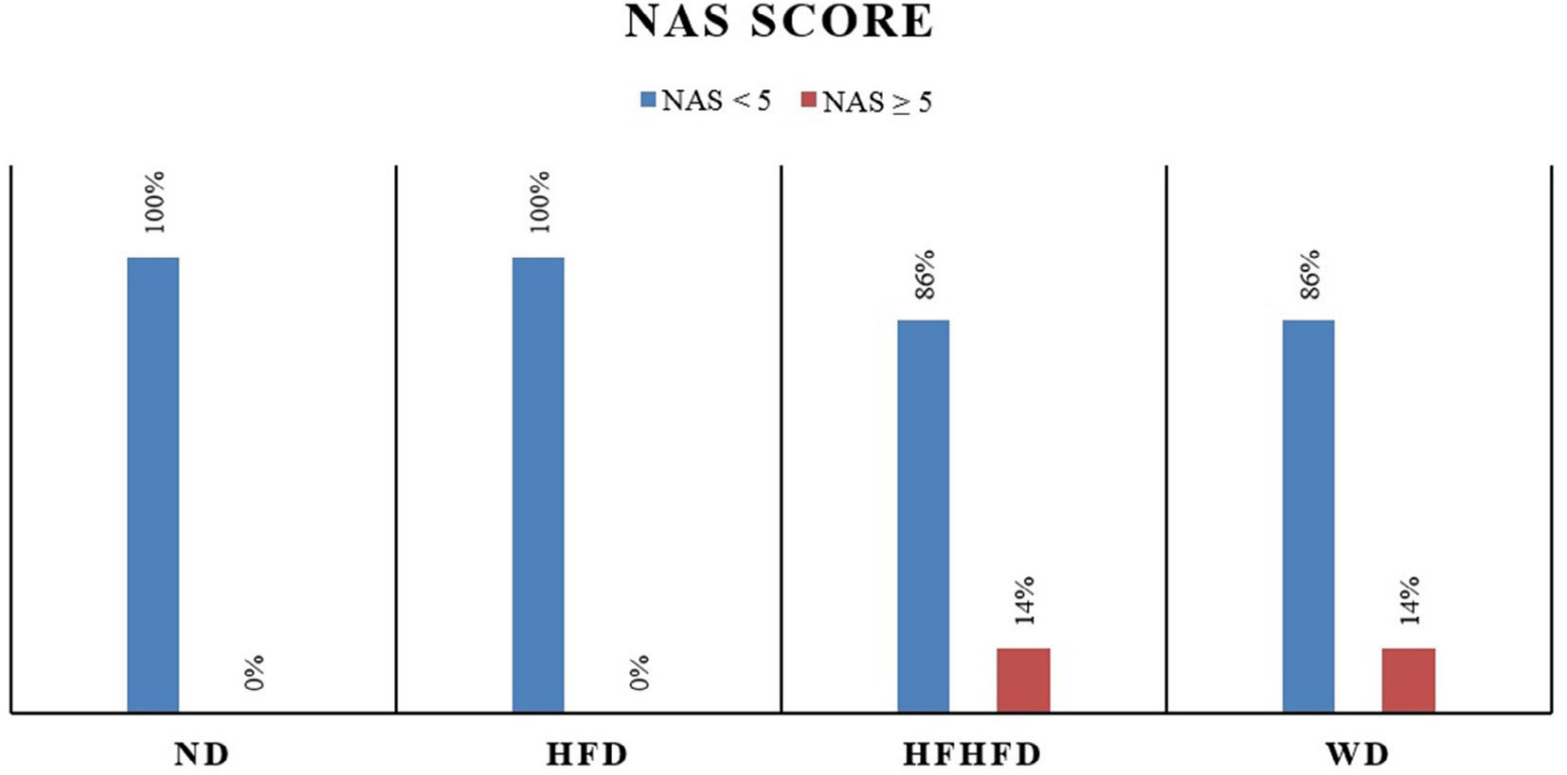
NAS percentages.

**Figure 9.**
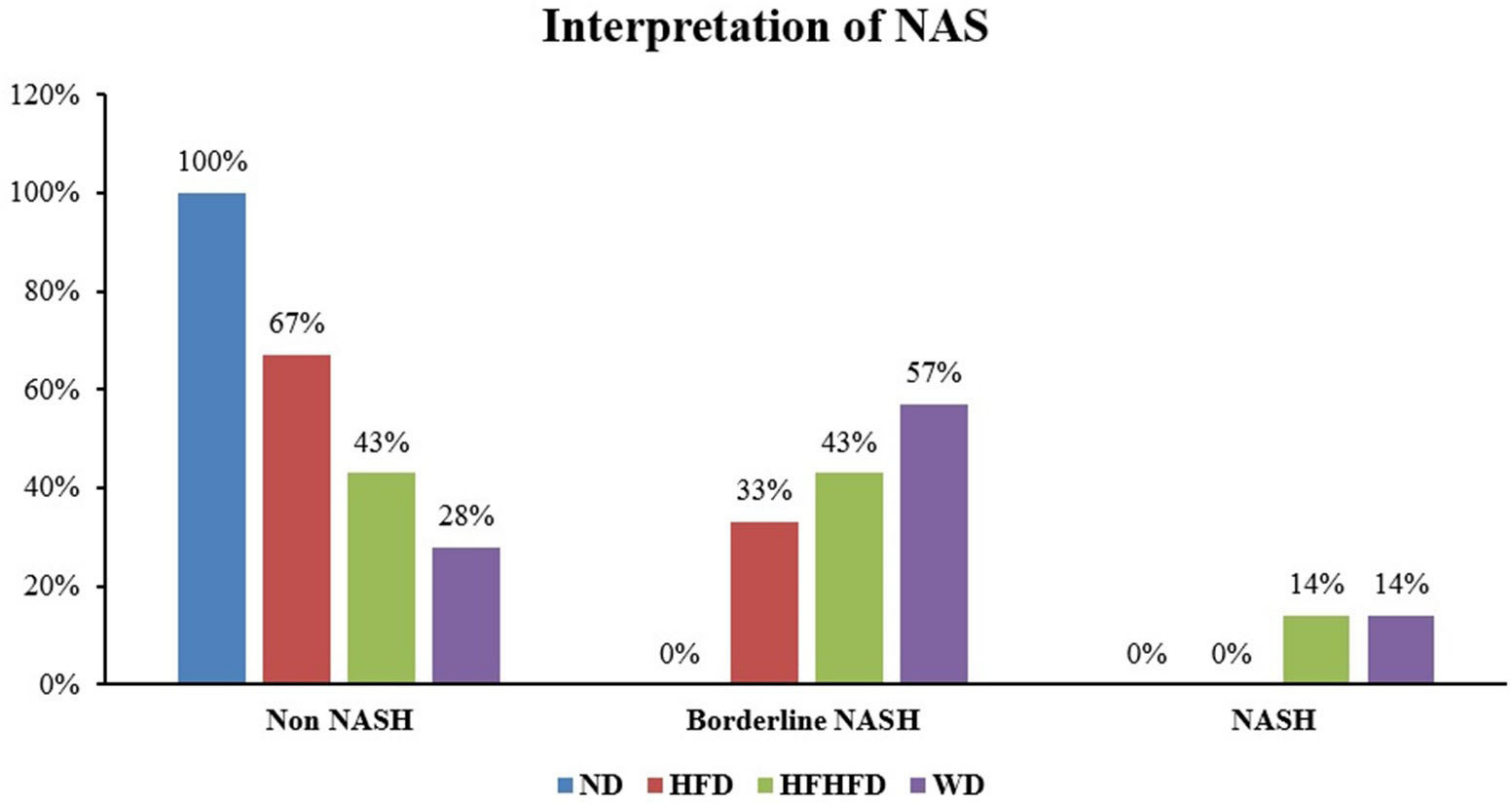
Interpretation of NAS.

**Figure 10.**
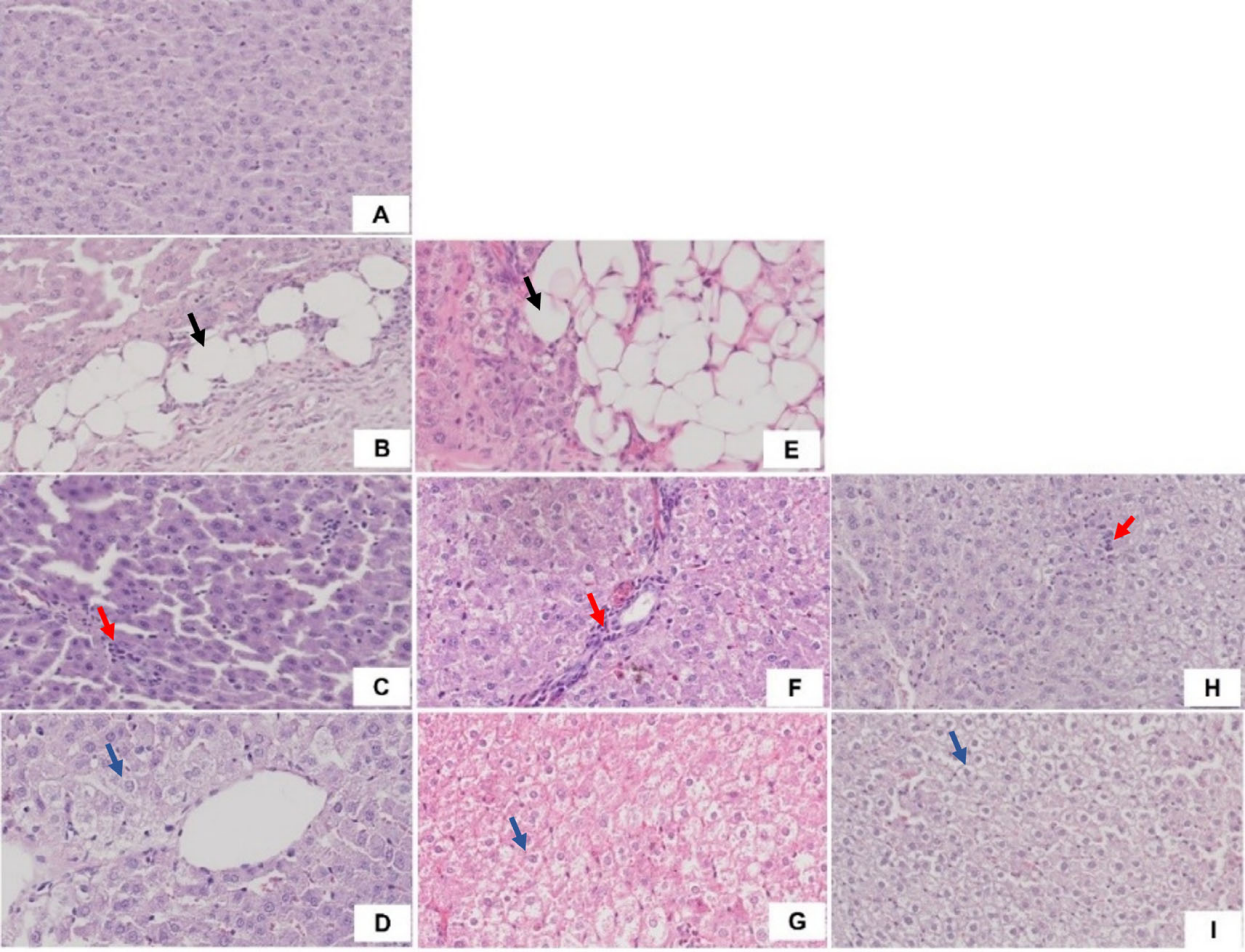
Liver histology. A). ND group; B). Steatosis in HFD group (black arrow); C). Lobular inflammation in HFD group (red arrow); D). Hepatocyte ballooning in HFD group (blue arrow); E). Steatosis in WD group (black arrow); F). Lobular inflammation in WD group (red arrow); G). Hepatocyte ballooning in WD group (blue arrow); H). Lobular inflammation in HFHFD group (red arrow); I). Hepatocyte ballooning in HFHFD group (blue arrow).

Liver histological analysis is presented in Figure 10. According to Table 2, the HFD group had the highest percentage of histological features, with lobular inflammation being the most common, only 33.33% developed hepatocyte ballooning. In contrast, in the WD and HFHFD groups, all rats had lobular inflammation, and most of them developed hepatocyte ballooning. Based on the NAS score, only the WD and HFHFD groups had NAS scores ≥5, and this was observed in the same percentage of rats ( Figure 8). The WD group had 57% of rats potentially experiencing NASH (borderline NAS), which was higher than the HFHFD group (43%) ( Figure 9).

**Table 2.** Liver histological findings.

Group	Steatosis	Lobular inflammation	Hepatocyte ballooning
ND (n = 7)	0%	0%	0%
HFD (n = 6)	16.67%	83.33%	33.33%
WD (n = 7)	14.28%	100%	85.71%
HFHD (n = 7)	0 %	100%	71.42%

## Discussion

The challenge in creating experimental animals arises when faced with intervention results that should reflect aspects of the complex etiopathogenesis of NAFLD in humans. Rats and mice have primarily been used as animal models of NAFLD. However, certain genetic animal models, such as *ob/ob* (leptin) mice or *db/db* (leptin receptor) mice can exhibit obesity phenotypes, ^15^^,^^16^ but do not reflect the etiology of obesity and related diseases as well as in humans as effectively. ^17^ Preclinical models of NASH are designed to mimic the same factors that trigger human disease, one of which is related to excessive calorie consumption. Various types of diet have been studied to induce NAFLD. Different diet compositions can alter the natural course of NAFLD, therefore it is important to discuss the impact of different types of diet on the development of NAFLD. A methionine and choline-deficient (MCD) diet is frequently used to induce NAFLD. Mice given MCD quickly develop steatosis and liver inflammation, followed by fibrosis within just 2-8 weeks of intervention. ^17^ However, the mice experienced weight loss, reduced blood sugar levels, and increased insulin sensitivity, which contrasts with the human condition. ^18^^,^^19^ Obesogenic diets, including various types of high-fat diets, are relatively more time-consuming in inducing NAFLD. However, this intervention can create an animal model of NASH with a spectrum of pathogenesis that more closely resembles humans. ^17^ Some obesogenic diets such as a high-fat and high-fructose diet can lead to liver fat accumulation and an increased risk of insulin resistance. ^20^ The Western diet, which contained high cholesterol, also induced pro-inflammatory and pro-fibrotic pathways in animal models of NASH. ^21^ Systemic low-grade inflammation, which has the potential to increase reactive oxygen species (ROS) and pro-oxidative stressors, is a hallmark of obesity. Obesity is associated with hyperglycemia and increased levels of free fatty acids (FFAs), which then induce lipotoxicity. ^22^ Increased FFAs and insulin resistance trigger hepatic steatosis. This condition has an impact on increasing hepatic lipase activity, responsible for hydrolyzing hepatic triglycerides and lipoprotein phospholipids. The degree of hepatic steatosis is positively correlated with hepatic lipase. ^23^ In our study, metabolic changes were found in the HFD, WD, and HFHFD groups. However, only rats on HFD and WD developed an obese phenotype by the end of the study, although the data were not significant. Triglyceride was found at the highest level in the WD group, while LDL was the highest in the HFD group. Hepatic lipase was found in excessive levels in the HFD group. A previous study proved that high-fat animals had significantly higher body weight than high-fructose animals. ^24^ Lee *et al.* stated that rats induced by high-fat and high-fat-high-fructose had significantly higher body weights than high-fructose only. ^25^

The adiposity index, an increase in body weight, and excessive fat accumulation are all signs of obesity. The fat and sugar composition in the WD may contribute to an increase in body weight by promoting the accumulation of abdominal fat mass and adiponectin expression in adipose tissue. Micronutrient composition in the WD could be the possible factor that affects a rat’s body weight gain. These results were in line with Bortolin et *al.* who concluded that the WD was the most effective diet to promote obesity in rats. Micronutrient content and diet palatability are factors that contribute to weight gain in rats. ^26^

Circulating inflammatory cytokines are primarily derived from adipose tissue. Through the inflammatory pathway, high levels of circulating inflammatory signals can cause insulin resistance and provide positive feedback that increases liver inflammation. By activating the c-Jun N-terminal kinase (JNK) and nuclear factor-kappa B (NF-κB) signaling pathways, obesity increases the production of pro-inflammatory cytokines like TNF-α and IL-6. ^27^ In our study, obese rats that were in the HFD and WD groups also developed higher levels of TNF-α and IL-6. These results were also consistent with other studies that revealed the effect of HFD rats and obese diabetic patients on TNF-α and IL-6. ^28^^,^^29^

Cholesterol and saturated fatty acids (SFAs) are examples of WD ingredients that are related to the inflammatory response in the immune system. ^30^ Insulin resistance in the WD model could cause hypertriglyceridemia and hypercholesterolemia, which induce lipotoxicity and hepatic steatosis. Accumulation of SFAs and cholesterol in the WD could cause hepatic oxidative stress by disruption of the glutathione system and superoxide dismutase (SOD) levels. Furthermore, oxidative stress may trigger the activation of NF-κB, mitogen-activated protein kinase (MAPK), and the JNK cascade, resulting in increasing several cytokines such as TNF-α and IL-6 in hepatocytes and Kupffer cells. ^31^

Metabolic changes and inflammatory conditions are closely related to the disruption of the intestinal barrier, leading to microbial dysbiosis. Gram-negative bacteria contain LPS, which in large quantities can induce an inflammatory response, leading to endotoxemia. The presence of LPS translocation that enters through the portal circulation can trigger the occurrence of repeated liver exposure, leading to liver injury. ^32^ Consuming high levels of fructose and fat was found to be strongly correlated with increased serum LPS levels, toll-like receptor 4 (TLR4) expression, as well as circulating cytokines. ^33^ A previous study confirmed the activation of the LPS–TLR4 pathway in obese rats induced by the HFHFD. ^34^ However, in our study, the results of LPS were not linear with other inflammatory cytokines (TNF-α and IL-6) and were theoretically inconsistent. This may caused by a short duration of intervention between groups.

Disruption of the gut microbiota also contributes to the production of SCFA such as acetic acid, propionic acid, and butyric acid. The lower levels of butyric acid were found significant in the HFHFD group (p = 0.03). Those previous studies supported our findings. Consumption of HFHFD has previously been proven to affect the homeostasis of gut microbiota and increase cholesterol levels, which is associated with an increased risk of intestinal diseases such as Crohn’s disease, ulcerative colitis, and colon cancer. ^35^^,^^36^ Some supporting evidence also revealed that the levels of butyric acid in patients with ulcerative colitis and Crohn’s disease were lower than a healthy control, ^37^ indicating that butyric acid might have a protective effect against inflammatory bowel disease. In our study, a HFHFD might contribute to the disruption of gut microbiota homeostasis and thereafter cause the impaired production of butyric acid, a type of SCFA produced by gut microbiota in the colon. ^38^ This indicates that a HFHFD might cause a decrease in butyric acid levels.

The theory of how a HFHFD affects the levels of butyric acid remains to be properly defined. However, some previous studies have proposed a possible mechanism. Briefly, a HFHFD may alter gut microbiota composition by reducing the *Megasphaera elsdenii* bacteria, a member of the *Firmicutes* group that can convert lactates into butyrate. In addition, after a HFHFD, it was reported that the beneficial *Bifidobacteria* and *Lactobacilli*, which interact with *Firmicutes* bacteria to produce butyric acid through cross-feeding, decreased in abundance. ^39^^,^^40^ This proposed theory may explain the mechanism by which an HFHFD impairs the production of butyric acid.

There is also growing interest in NAFLD pathomechanism related to the gut-brain-liver axis. Nutritional imbalance induced by obesogenic diet promoted microbial dysbiosis. It stimulated the intestinal endocrine (L cell) to release GLP-1 which acted in the vagus nerve. Gut-vagal afferent nerve was continually activated during inflammation. It stimulates the brain to regulate insulin sensitivity, glucose production, and fatty acid oxidation. ^41^ Previous studies proved that psychological stress and HFHFD feeding promoted alteration in intestinal tight junction proteins, increased in insulin resistance and plasma cholesterol, and impacted the RNA expression of inflammatory factors in the hippocampus. ^42^ WD consumption stimulated endotoxemia and promoted neuroinflammation and cognitive dysfunction, and also impaired insulin sensitivity. ^43^ Another study proved that a shorter duration of WD feeding induced brain neuroinflammation in mice, while for longer duration promoted advanced hypercholesterolemia and NAFLD. ^44^

The duration, type of diet, and genetic factors all play a role in the development of NAFLD-associated liver histology. ^45^ In our study, steatosis, lobular inflammation, and hepatocyte ballooning were observed in the HFD and WD groups, while only lobular inflammation and hepatocyte ballooning were found in the HFHFD group. NAS scores ≥ 5 were found in the WD and HFHFD groups. Although both WD and HFHFD met NASH criteria based on NAS scores, a higher percentage of borderline NASH was found in the WD group. A previous study stated that a diet-induced animal model of non-alcoholic fatty liver disease (DIAMOND) induced by a western diet along with a high fructose solution (42% fat, 0.1% cholesterol, high fructose/glucose water) developed steatosis, steatohepatitis, fibrosis progressive, and hepatocellular carcinoma (HCC) after 52 weeks of intervention. ^46^ Another study evaluated the effects of different diets (WD, cafeteria diet, and HFD) for 18 weeks of intervention and found that the WD induced obesity and insulin resistance increased the leptin/adiponectin ratio, increased TNF-α and IL-6, and had the highest steatosis scores among the other groups. ^26^

This research has several limitations. First, it is essential to consider total cholesterol, high-density lipoprotein (HDL), and body fat index for a more comprehensive understanding of their potential role in the metabolic aspects after dietary intervention. Second, insulin resistance has a role in steatosis development and can trigger elevated hepatic lipase activity. Although this study measured hepatic lipase levels, it did not assess insulin levels. Third, it is need to examine the gut microbiota profile and identify the significant microorganisms contributing to each intervention. This can help develop more precise treatments for the future. Further research should also compare and evaluate the impact of different durations of food consumption.

## Conclusions

In summary, different types of high-fat diets influence metabolic markers, inflammatory markers and dysbiosis related to NAFLD. The HFD group induces significant liver inflammation but does not produce NASH, whereas the WD and HFHFD progress to NASH. In terms of NASH development histologically, WD is better than HFHFD. So, among high-fat diet types, the WD is the most appropriate diet to induce NASH in rats.

## Data availability

### Underlying data

Dryad. Data of Multiple Different High-Fat Diets. https://doi.org/10.5061/dryad.np5hqbzxx. ^47^

This project contains the following underlying data:

• **Data file 1: Data of Normal Diet Conditioning**

Data files contain all measurements conducted during the ND conditioning of rats, including body weight, biochemical analysis using blood samples, SCFA analysis using feces, NAS analysis through liver histology, mean, Q1, Q3, and deviation standard of each measurement.

• **Data file 2: Data of High Fat Diet Conditioning**

Measurements conducted during the HFD conditioning of rats, including body weight, biochemical analysis using blood samples, SCFA using feces, NAS analysis through liver histology, mean, Q1, Q3, and deviation standard of each measurement.

• **Data file 3: Data of Western Diet Conditioning**

Measurements conducted during the WD conditioning of rats, including body weight, biochemical analysis using blood samples, SCFA analysis using feces, NAS analysis through liver histology, mean, Q1, Q3, and deviation standard of each measurement.

• **Data file 4: Data of High Fat High Fructose Diet Conditioning**

Measurements conducted during the HFHFD conditioning of rats, including the body weight, biochemical analysis using blood samples, SCFA analysis using feces, NAS analysis through liver histology, mean, Q1, Q3, and deviation standard of each measurement.

• **README.md**

README.md is a note that contains information and a summary of the dataset, as well as an explanation of the variables under study, the abbreviations, and units of measurement.

• **Related Work – Supplemental Information**

This project consists of the 10 supplemental figures, the document of SCFA analysis using Shimadzu, and the full ARRIVE author checklist. Data are available under the terms of the Creative Commons Attribution 4.0 International. https://doi.org/10.5281/zenodo.7583400. ^48^

Data are available under the terms of the Dryad’s Term of Service and under the terms of the Creative Commons Zero “No rights reserved” data waiver (CC0 1.0 Public domain dedication).

## References

[1] Mitra S De A Chowdhury A : Epidemiology of non-alcoholic and alcoholic fatty liver diseases. *Transl Gastroenterol Hepatol.* 2020;5(16):16–17. 10.21037/tgh.2019.09.08 32258520 PMC7063528

[2] Omagari K Suzuta M Taniguchi A : A non-obese, diet-induced animal model of nonalcoholic steatohepatitis in Wistar/ST rats compared to Sprague-Dawley rats. *Clin Nutr Exp.* 2020;30:1–14. 10.1016/j.yclnex.2020.03.001

[3] Chalasani N Younossi Z Lavine JE : The diagnosis and management of nonalcoholic fatty liver disease: Practice guidance from the American Association for the Study of Liver Diseases. *Hepatology.* 2018;67(1):328–357. 10.1002/hep.29367 28714183

[4] Jarvis H Craig D Barker R : Metabolic risk factors and incident advanced liver disease in non-alcoholic fatty liver disease (NAFLD): A systematic review and meta-analysis of population-based observational studies. *PLoS Med.* 2020;17(4):e1003100. 10.1371/journal.pmed.1003100 32353039 PMC7192386

[5] Salehi A Sadat S Beigrezaei S : Dietary patterns and risk of non - alcoholic fatty liver disease. *BMC Gastroenterol.* 2021;21(41):1–12. 33509112 10.1186/s12876-021-01612-z PMC7844966

[6] Paik JM Henry L De Avila L : Mortality Related to Nonalcoholic Fatty Liver Disease Is Increasing in the United States. *Hepatol Commun.* 2019;3(11):1459–1471. 10.1002/hep4.1419 31701070 PMC6824058

[7] Ando Y Jou JH : Nonalcoholic Fatty Liver Disease and Recent Guideline Updates. *Clin. Liver Dis.* 2021;17(1):23–28. 10.1002/cld.1045 33552482 PMC7849298

[8] Handayani D Meyer BJ Chen J : A High-Dose Shiitake Mushroom Increases Hepatic Accumulation of Triacylglycerol in Rats Fed a High-Fat Diet: Underlying Mechanism. *Nutrients.* 2014;6:650–662. 10.3390/nu6020650 24566434 PMC3942724

[9] Stephenson K Kennedy L Hargrove L : Updates on Dietary Models of Nonalcoholic Fatty Liver Disease: Current Studies and Insights. *Gene Expr.* 2017;18(1):5–17. 10.3727/105221617X15093707969658 29096730 PMC5860971

[10] Linsenmeier RA Beckmann L Dmitriev AV : Intravenous ketamine for long term anesthesia in rats. *Heliyon.* 2020;6(12):e05686. 10.1016/j.heliyon.2020.e05686 33367124 PMC7749388

[11] Savari F Mard SA Badavi M : A new method to induce nonalcoholic steatohepatitis (NASH) in mice. *BMC Gastroenterol.* 2019;19(1):125. 10.1186/s12876-019-1041-x 31307427 PMC6632212

[12] Fan Y Xiong W Li J : Mechanism of TangGanJian on nonalcoholic fatty liver disease with type 2 diabetes mellitus. *Pharm Bio.* 2018;56(1):567–572. 10.1080/13880209.2018.1504972 30460863 PMC6249541

[13] Kamil RZ Murdiati A Juffrie M : Gut microbiota and short-chain fatty acid profile between normal and moderate malnutrition children in Yogyakarta. Microorganisms. 2021;9(1):1–15. 10.3390/microorganisms9010127 33430510 PMC7826765

[14] Lee G You HJ Bajaj JS : Distinct signatures of gut microbiome and metabolites associated with significant fibrosis in non-obese NAFLD. *Nat Commun.* 2020;11(1):1–13. 10.1038/s41467-020-18754-5 33020474 PMC7536225

[15] Mayer J Bates MW Dickie MM : Hereditary Diabetes in Genetically Obese Mice. *Science.* 1951;113(2948):746–747. 10.1126/science.113.2948.746 14854871

[16] Hummel KP Dickie MM Coleman DL : Diabetes, a New Mutation in the Mouse. *Science.* 1966;153(3740):1127–1128. 10.1126/science.153.3740.1127 5918576

[17] Jahn D Kircher S Hermanns HM : Animal models of NAFLD from a hepatologist’s point of view. *Biochim Biophys Acta Mol Basis Dis.* 2019;1865(5):943–953. 10.1016/j.bbadis.2018.06.023 29990551

[18] Rinella ME Green RM : The methionine-choline deficient dietary model of steatohepatitis does not exhibit insulin resistance. *J Hepatol.* 2004;40(1):47–51. 10.1016/j.jhep.2003.09.020 14672613

[19] Rizki G Arnaboldi L Gabrielli B : Mice fed a lipogenic methionine-choline-deficient diet develop hypermetabolism coincident with hepatic suppression of SCD-1. *J Lipid Res.* 2006;47(10):2280–2290. 10.1194/jlr.M600198-JLR200 16829692

[20] Buzzetti E Pinzani M Tsochatzis EA : The multiple-hit pathogenesis of non-alcoholic fatty liver disease (NAFLD). *Metabolism.* 2016;65(8):1038–1048. 10.1016/j.metabol.2015.12.012 26823198

[21] Henkel J Coleman CD Schraplau A : Induction of steatohepatitis (NASH) with insulin resistance in wild-type B6 mice by a western-type diet containing soybean oil and cholesterol. Mol Med. 2017;23:70–82. 10.2119/molmed.2016.00203 28332698 PMC5429885

[22] Duan Y Zeng L Zheng C : Inflammatory Links Between High Fat Diets and Diseases. *Front Immunol.* 2018;9(2649):1–10. 10.3389/fimmu.2018.02649 30483273 PMC6243058

[23] Cedo L Santos D Rivas-urbina A : Human hepatic lipase overexpression in mice induces hepatic steatosis and obesity through promoting hepatic lipogenesis and white adipose tissue lipolysis and fatty acid uptake. *PLoS One.* 2017;12(12):e0189834–14. 10.1371/journal.pone.0189834 29244870 PMC5731695

[24] Woodie L Blythe S : The differential effects of high-fat and high-fructose diets on physiology and behavior in male rats. *Nutr Neurosci.* 2017;21(5):328–336. 10.1080/1028415X.2017.1287834 28195006

[25] Lee JS Jun DW Kim EK : Histologic and metabolic derangement in high-fat, high-fructose, and combination diet animal models. *Sci World J.* 2015;2015(306326):1–9. 10.1155/2015/306326 26090514 PMC4451512

[26] Bortolin RC Vargas AR Gasparotto J : A new animal diet based on human Western diet is a robust diet-induced obesity model: Comparison to high-fat and cafeteria diets in term of metabolic and gut microbiota disruption. *Int J Obes.* 2018;42(3):525–534. 10.1038/ijo.2017.225 28895587

[27] Chen Z Yu R Xiong Y : A vicious circle between insulin resistance and inflammation in nonalcoholic fatty liver disease. *Lipids Health Dis.* 2017;16(1):203–9. 10.1186/s12944-017-0572-9 29037210 PMC5644081

[28] Goyal R Faizy AF Siddiqui SS : Evaluation of TNF-α and IL-6 Levels in Obese and Non-obese Diabetics: Pre- and Postinsulin Effects. *N* *Am J Med Sci.* 2012;4(4):180–184. 10.4103/1947-2714.94944 22536561 PMC3334258

[29] Adegbola PI Fadahunsi OS Ajilore BS : Combined ginger and garlic extract improves serum lipid profile, oxidative stress markers and reduced IL-6 in diet induced obese rats. *Obes Med.* 2021;23:100336. 10.1016/j.obmed.2021.100336

[30] Christ A Lauterbach M Latz E : Western Diet and the Immune System: An Inflammatory Connection. *Immunity.* 2019;51(5):794–811. 10.1016/j.immuni.2019.09.020 31747581

[31] Sabir U Muhammad H Ullah A : Downregulation of hepatic fat accumulation, inflammation and fibrosis by nerolidol in purpose built western-diet-induced multiple-hit pathogenesis of NASH animal model. *Biomed Pharmacother.* 2022;150(112956):112956. 10.1016/j.biopha.2022.112956 35447548

[32] An L Wirth U Koch D : The Role of Gut-Derived Lipopolysaccharides and the Intestinal Barrier in Fatty Liver Diseases. *J Gastrointest Surg.* 2022;26(3):671–683. 10.1007/s11605-021-05188-7 34734369 PMC8926958

[33] Lambertz J Weiskirchen S Landert S : Fructose: A dietary sugar in crosstalk with microbiota contributing to the development and progression of non-alcoholic liver disease. *Front Immunol.* 2017;8(1159). 10.3389/fimmu.2017.01159 28970836 PMC5609573

[34] Li KP Yuan M Wu YL : A High-Fat High-Fructose Diet Dysregulates the Homeostatic Crosstalk Between Gut Microbiome, Metabolome, and Immunity in an Experimental Model of Obesity. *Mol Nutr Food Res.* 2022;66(7):e2100950. 10.1002/mnfr.202100950 35072983

[35] Hold GL Smith M Grange C : Role of the gut microbiota in inflammatory bowel disease pathogenesis: What have we learnt in the past 10 years? *World J Gastroenterol.* 2014;20(5):1192–1210. 10.3748/wjg.v20.i5.1192 24574795 PMC3921503

[36] Sánchez-Alcoholado L Ramos-Molina B Otero A : The role of the gut microbiome in colorectal cancer development and therapy response. *Cancers (Basel).* 2020;12(6):1–29. 10.3390/cancers12061406 32486066 PMC7352899

[37] Tang X Li X Wang Y : Butyric Acid Increases the Therapeutic Effect of EHLJ7 on Ulcerative Colitis by Inhibiting JAK2/STAT3/SOCS1 Signaling Pathway. *Front Pharmacol.* 2020;10(1553):1–10. 10.3389/fphar.2019.01553 PMC6987075 32038241

[38] Onyszkiewicz M Gawrys-kopczynska M Konopelski P : Butyric acid, a gut bacteria metabolite, lowers arterial blood pressure via colon-vagus nerve signaling and GPR41/43 receptors. *Pflugers Arch.* 2019;471:1441, 31728701–1453. 10.1007/s00424-019-02322-y 31728701 PMC6882756

[39] Horne RG Yu Y Zhang R : High Fat-High Fructose Diet-Induced Changes in the Gut Microbiota Associated with Dyslipidemia in. *Nutrients.* 2020;12(11):3557. 10.3390/nu12113557 33233570 PMC7699731

[40] Markowiak-kope P Śliżewska K : The Effect of Probiotics on the Production of Short-Chain Fatty Acids by Human Intestinal Microbiome. *Nutrients.* 2020;12(4):1107. 10.3390/nu12041107 32316181 PMC7230973

[41] Zhang Q Xing W Wang Q : Gut microbiota–mitochondrial inter-talk in non-alcoholic fatty liver disease. Front Nutr. 2022;9: 934113. 10.3389/fnut.2022.934113 36204383 PMC9530335

[42] Rodrigues EM de S Bekhbat M Houser MC : Chronic psychological stress and high-fat high-fructose diet disrupt metabolic and inflammatory gene networks in the brain, liver, and gut and promote behavioral deficits in mice. *Brain Behav Immun.* 2017;59:158–172. 10.1016/j.bbi.2016.08.021 27592562 PMC5154856

[43] Noble EE Hsu TM Kanoski SE : Gut to brain dysbiosis: Mechanisms linking western diet consumption, the microbiome, and cognitive impairment. *Front Behav Neurosci.* 2017;11(9). 10.3389/fnbeh.2017.00009 PMC5277010 28194099

[44] Wiȩckowska-Gacek A Mietelska-Porowska A Chutorański D : Western Diet Induces Impairment of Liver-Brain Axis Accelerating Neuroinflammation and Amyloid Pathology in Alzheimer’s Disease. *Front Aging Neurosci.* 2021;13(654509). 10.3389/fnagi.2021.654509 33867971 PMC8046915

[45] Kucera O Cervinkova Z : Experimental models of non-alcoholic fatty liver disease in rats. *World J Gastroenterol.* 2014;20(26):8364–8376. 10.3748/wjg.v20.i26.8364 25024595 PMC4093690

[46] Asgharpour A Cazanave SC Pacana T : A diet-induced animal model of non-alcoholic fatty liver disease and hepatocellular cancer. *J Hepatol.* 2016;65(3):579–588. 10.1016/j.jhep.2016.05.005 27261415 PMC5012902

[47] Mustika S Santosaningsih D Handayani D : Data of Multiple Different High-Fat Diets. *Dryad.* 2023. 10.5061/dryad.np5hqbzxx PMC11369591 39229607

[48] Mustika S Santosaningsih D Handayani D : Data of multiple different high-fat diets. *Zenodo.* 2023. 10.5281/zenodo.7583400 PMC11369591 39229607

