# Supplementary material for: Impact of multiple different high-fat diets on metabolism, inflammatory markers, dysbiosis, and liver histology: study on NASH rat model induced diet
Source: F1000Res. 2023 Dec 12;12:180. Originally published 2023 Feb 15. [Version 2] doi: 10.12688/f1000research.129645.2 (PMC11369591; doi:10.12688/f1000research.129645.2)
Supplement: Peer review history

# Reviewer report: Reviewer response for version 2

**DOI:** 10.5256/f1000research.159829.r229533

**Published:** 2024-09-02

**Authors:** Pabulo Henrique Rampelotto

**Affiliations:** 1 Graduate Program in Biological Sciences: Pharmacology and Therapeutics, Universidade Federal do Rio Grande do Sul, Porto Alegre, Brazil

The authors have made significant changes in the manuscript following our suggestions and the revised article is suitable in its current form.

Is the work clearly and accurately presented and does it cite the current literature?

Yes

If applicable, is the statistical analysis and its interpretation appropriate?

Yes

Are all the source data underlying the results available to ensure full reproducibility?

Partly

Is the study design appropriate and is the work technically sound?

Yes

Are the conclusions drawn adequately supported by the results?

Yes

Are sufficient details of methods and analysis provided to allow replication by others?

Yes

Reviewer Expertise:

Microbiome; molecular microbiology; metagenomics; omics; next-generation sequencing; microbial ecology; bioinformatics; biotechnology; system biology; grand challenges

I confirm that I have read this submission and believe that I have an appropriate level of expertise to confirm that it is of an acceptable scientific standard.

# Reviewer report: Reviewer response for version 1

**DOI:** 10.5256/f1000research.142342.r163637

**Published:** 2023-04-12

**Authors:** Endang Sutriswati Rahayu

**Affiliations:** 1 Department of Food and Agricultural Product Technology, Faculty of Agricultural Technology, Universitas Gadjah Mada, Yogyakarta, Indonesia

The paper discussed different high-fat diets of animal models with NASH which may affect the metabolism, inflammatory markers, dysbiosis and liver histology. Overall, the study reports interesting results. However, some revisions are needed on how the results are presented.

 The statistical analysis, as I have filled out as partly appropriate, needs to be revised on how it is presented. In Table 1, the baseline characteristics and biochemical parameters of rats are shown. The term baseline characteristics are used for data before the intervention is done, yet the table seems to show the data after the intervention is conducted. If the data shown is after the intervention, then it is advised to change the title which also mentions the intervention period.

 The author should mention which data is different after posthoc comparison in Table 1 (e.g., with superscript letters that can be found using the cld function in R).

 Please refrain from using personal pronouns (i.e., We) in writing scientific papers.

Is the work clearly and accurately presented and does it cite the current literature?

Yes

If applicable, is the statistical analysis and its interpretation appropriate?

Partly

Are all the source data underlying the results available to ensure full reproducibility?

Yes

Is the study design appropriate and is the work technically sound?

Yes

Are the conclusions drawn adequately supported by the results?

Yes

Are sufficient details of methods and analysis provided to allow replication by others?

Yes

Reviewer Expertise:

Gut microbiome, food microbiology

I confirm that I have read this submission and believe that I have an appropriate level of expertise to confirm that it is of an acceptable scientific standard.

# Response

**Published:** 2023-12-05

**Authors:** Syifa Mustika

**Affiliations:** Universitas Brawijaya, Indonesia

1. In Table 1, the baseline characteristics and biochemical parameters of rats are shown. The term baseline characteristics are used for data before the intervention is done, yet the table seems to show the data after the intervention is conducted. If the data shown is after the intervention, then it is advised to change the title which also mentions the intervention period. The author should mention which data is different after posthoc comparison in Table 1

 - The title in Table 1 is revised to “Comparison of various diets induction in rats after 12 weeks of intervention”.

 The posthoc test result was presented in the graph and mentioned in the subsection below:

- Comparison of metabolic parameters of rats (in Figure 2)
- Comparison of inflammatory and liver injury of rats (in Figure 3, Figure 4, and Figure 5)

Comparison of microbial dysbiosis (in Figure 6 and Figure 7)

 2. Please refrain from using personal pronouns (i.e., We) in writing scientific papers.

 - Thank you for your suggestion. We have made changes to the manuscript

# Reviewer report: Reviewer response for version 1

**DOI:** 10.5256/f1000research.142342.r165523

**Published:** 2023-03-24

**Authors:** Pabulo Henrique Rampelotto, Elisa Carolina Lange

**Affiliations:** 1 Graduate Program in Biological Sciences: Pharmacology and Therapeutics, Universidade Federal do Rio Grande do Sul, Porto Alegre, Brazil; 2 Graduate Program in Gastroenterology and Hepatology, Universidade Federal do Rio Grande do Sul, Porto Alegre, State of Rio Grande do Sul, Brazil

Overall, the manuscript provides a comprehensive overview of the factors involved in the development of non-alcoholic fatty liver disease (NAFLD) and its progression to non-alcoholic steatohepatitis (NASH). The authors cover a wide range of topics, including the role of diet, inflammation, oxidative stress, mitochondrial dysfunction, dysbiosis of gut microbiota, and genetic factors in the pathogenesis of NAFLD/NASH.

 The manuscript also includes data from an animal study investigating the effects of different diets on metabolic changes and inflammatory conditions. The authors provide a detailed analysis of the results, highlighting the differences in body weight, lipid profiles, and inflammatory cytokine levels between the high-fat diet (HFD), Western diet (WD), and high-fat high-fructose diet (HFHFD) groups. However, there are several areas where the manuscript could be improved.     

          

  Firstly, the manuscript lacks a clear research question or hypothesis that the study aims to answer. Without a clear research question, the manuscript can come across as a collection of unrelated findings. A clearly stated research question would provide a framework for the manuscript and help readers understand the significance of the study.

 Second, the authors could improve the clarity of the writing. Some of the sentences are overly complex and difficult to follow, making it hard for readers to understand the main points. Simplifying the language and breaking down complex ideas into smaller, more manageable pieces would help readers engage with the manuscript.

 Third, the authors should provide more detail on the methods used in the animal study. For example, the authors do not mention the number of animals used in each group, the euthanasia process (did the rats fast before euthanasia?), etc. Providing this information would help readers understand the study design and assess the reliability of the results.

 Fourth, several relevant parameters for NASH models were not measured, like cholesterol, insulin, microbiota, as well as other key lipid metabolites. This should be clearly discussed as the limitations of the study.

 Fifth, no discussion is provided regarding other well-established nutritional models of NASH (e.g., high-fat + choline-deficient diet). On this aspect, authors should also provide a discussion on the challenges encountered in NAFLD animal models, i.e., how to establish the optimal animal model which could mirror human disease by providing the same pathological triggers, as well as reproducible mechanisms of progression towards NASH and its complications.

  Finally, while the manuscript provides a good overview of the current understanding of NAFLD/NASH, there are several recent developments in the field that the authors could incorporate into the discussion. For example, recent studies have identified novel genetic variants associated with NAFLD/NASH, and there is growing interest in the role of the gut-brain axis in the pathogenesis of these conditions. Including these recent findings would help the manuscript stay up-to-date and relevant. 

     

 In conclusion, the manuscript provides a comprehensive overview of the factors involved in the development of NAFLD/NASH and presents data from an animal study investigating the effects of different diets on metabolic changes and inflammatory conditions. However, the authors could improve the manuscript by providing a clear research question, improving the clarity of the writing, providing more detail on the methods used in the animal study, and better discussing the limitations of the study in comparison with other robust experimental models of NAFLD/NASH, as well as incorporating recent developments in the field into the discussion.

Is the work clearly and accurately presented and does it cite the current literature?

Yes

If applicable, is the statistical analysis and its interpretation appropriate?

Yes

Are all the source data underlying the results available to ensure full reproducibility?

Partly

Is the study design appropriate and is the work technically sound?

Yes

Are the conclusions drawn adequately supported by the results?

Yes

Are sufficient details of methods and analysis provided to allow replication by others?

Yes

Reviewer Expertise:

Microbiome; molecular microbiology; metagenomics; omics; next-generation sequencing; microbial ecology; bioinformatics; biotechnology; system biology; grand challenges

We confirm that we have read this submission and believe that we have an appropriate level of expertise to confirm that it is of an acceptable scientific standard, however we have significant reservations, as outlined above.

# Response

**Published:** 2023-12-05

**Authors:** Syifa Mustika

**Affiliations:** Universitas Brawijaya, Indonesia

1. the manuscript lacks a clear research question or hypothesis that the study aims to answer. Without a clear research question, the manuscript can come across as a collection of unrelated findings.

 - We have made changes in the manuscript: This study aims to compare and determine the most representative diet for inducing NASH in the *Rattus norvegicus* Wistar strain. In order to ascertain whether the rats have developed NASH, many parameters, including metabolic, inflammatory, and liver damage, microbial dysbiosis, and liver histology, were investigated.

 2. the authors could improve the clarity of the writing. Some of the sentences are overly complex and difficult to follow, making it hard for readers to understand the main points.

 -Thank you for the suggestion, we have made changes on the manuscript.

 3. the authors should provide more detail on the methods used in the animal study. For example, the authors do not mention the number of animals used in each group, the euthanasia process (did the rats fast before euthanasia?), etc. Providing this information would help readers understand the study design and assess the reliability of the results.

 - Thank you for your suggestion. The number of animals used in each group was mentioned in the Figure 1 research flow. But, to clarify this study, we mentioned the number of animals used in each group in the method section, animals, and diet sub-section.

 - The euthanasia process is mentioned in the animals and diet subsection and revised below:

 After the last diet intervention administration, the rats fasted for 12 hours but consumed water freely. After that, all groups were euthanized with ketamine–xylazine intravenously to relieve pain on the same day before surgery was performed. The blood serum and liver were taken for further testing.

 4. several relevant parameters for NASH models were not measured, like cholesterol, insulin, microbiota, as well as other key lipid metabolites. This should be clearly discussed as the limitations of the study.

 - Thank you for your suggestion. We have made changes to the manuscript in the limitation section

 5. there are several recent developments in the field that the authors could incorporate into the discussion. For example, recent studies have identified novel genetic variants associated with NAFLD/NASH, and there is growing interest in the role of the gut-brain axis in the pathogenesis of these conditions. Including these recent findings would help the manuscript stay up-to-date and relevant.

 - Thank you for your suggestion. We have made changes to the manuscript in the discussion section

 6. the authors could improve the manuscript by providing a clear research question, improving the clarity of the writing, providing more detail on the methods used in the animal study, and better discussing the limitations of the study in comparison with other robust experimental models of NAFLD/NASH, as well as incorporating recent developments in the field into the discussion.

 - Thank you for your suggestion. We have made changes to the manuscript.
